# Superconducting nonlinear transport in optically driven high-temperature K_3_C_60_

**DOI:** 10.1038/s41467-023-42989-7

**Published:** 2023-11-09

**Authors:** E. Wang, J. D. Adelinia, M. Chavez-Cervantes, T. Matsuyama, M. Fechner, M. Buzzi, G. Meier, A. Cavalleri

**Affiliations:** 1 Max Planck Institute for the Structure and Dynamics of Matter, Hamburg, Germany; 2 Department of Physics, Clarendon Laboratory, University of Oxford, Oxford, UK

**Keywords:** Superconducting properties and materials, Optoelectronic devices and components

## Abstract

Optically driven quantum materials exhibit a variety of non-equilibrium functional phenomena, which to date have been primarily studied with ultrafast optical, X-Ray and photo-emission spectroscopy. However, little has been done to characterize their transient electrical responses, which are directly associated with the functionality of these materials. Especially interesting are linear and nonlinear current-voltage characteristics at frequencies below 1 THz, which are not easily measured at picosecond temporal resolution. Here, we report on ultrafast transport measurements in photo-excited K_3_C_60_. Thin films of this compound were connected to photo-conductive switches with co-planar waveguides. We observe characteristic nonlinear current-voltage responses, which in these films point to photo-induced granular superconductivity. Although these dynamics are not necessarily identical to those reported for the powder samples studied so far, they provide valuable new information on the nature of the light-induced superconducting-like state above equilibrium T_c_. Furthermore, integration of non-equilibrium superconductivity into optoelectronic platforms may lead to integration in high-speed devices based on this effect.

## Abstract (web summary)

The authors report ultrafast transport measurements on the photo-excited superconducting state in K_3_C_60_. They observe characteristic superconducting nonlinear current-voltage responses.

## Introduction

**Fig. 1:**
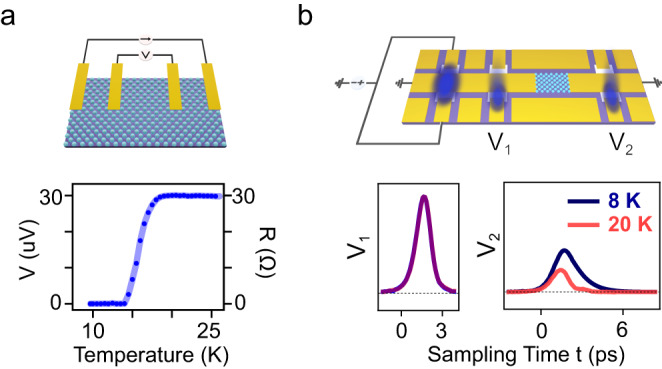
Conventional DC four-point transport measurement and on-chip ultrafast transport measurement. **a** Upper: schematic of a DC four-point transport measurement on a K_3_C_60_ thin film. Lower: measured resistance versus temperature of the K_3_C_60_ thin film with 1 µA current bias. **b** Upper: schematic of on-chip ultrafast transport device. The MBE-grown K_3_C_60_ thin film (cyan) and three pairs of photo-conductive switches (white) were incorporated within a coplanar waveguide (yellow) on a sapphire substrate (purple). Ultrashort electrical pulses were launched by illuminating laser pulses onto the left pair of photo-conductive switches, which were simultaneously biased by a voltage source. The launched pulse V_1_(t) was sampled by illuminating one switch of the middle pair and by changing the mutual delay between the launching and sampling laser pulses. Similarly, the transmitted pulse V_2_(t) was sampled with one switch of the right pair. Lower: V_1_(t) and V_2_(t) measured at 8 K and 20 K. Both measurements were normalized by the peak value of V_1_(t).

The electrical conductivity of materials is frequently characterized by static electrical transport measurements. Transport measurements have led to many discoveries, such as superconductivity^1^, the metal-insulator transition^2^, the charge density wave transition^3^, and the quantum Hall effect^4^. Yet, electrical measurements are not easily achieved in transient phases of matter, such as photo-induced superconductors^5–14^, Floquet states^15–19^, and transient topological phases^20,21^. On the one hand, conventional four-point transport methods (Fig. 1a) can only operate at quasi-DC frequency. On the other hand, optical time domain terahertz probes are only applicable to large samples, as determined by the corresponding diffraction limits.

Recently, ultrafast Hall transport measurements have been demonstrated in photo-excited graphene^16^. However, nonlinear electrical characteristics^22–26^ have remained largely unexplored. Here, photo-conductive switches^27^ and Terahertz (THz) waveguides were used to generate, transport, and detect electrical pulses of only ~1 picosecond duration. These electrical pulses were confined to deeply sub-wavelength length scales in spectral ranges from 50 GHz to 1 THz^16,28–32^, and used to measure the nonlinear transport response in the transient superconducting state of K_3_C_60_.

A number of recent experiments have shown that K_3_C_60_ can be manipulated with coherent optical drives, and have revealed signatures of optically induced superconductivity at temperatures above the equilibrium T_c_^7–9,14^. This has been shown using optical pump-probe measurements with superconducting-like optical features. This assignment was further supported by measurements performed under pressure^8^. Furthermore, electrical transport measurements showed a vanishingly small electrical resistance, also compatible with photo-induced superconductivity^9^. However, although all these observations are highly suggestive of a non-equilibrium superconducting state induced by light, important indicators like Meissner diamagnetism and critical current responses have not been reported yet. The newly developed ultrafast transport platform here is ideal for probing the critical current response at ultrafast timescales.

## Results and discussion

Thin films of K_3_C_60_ were grown on sapphire substrates by molecular beam epitaxy (MBE), and optimized to yield a superconducting phase with T_c_ = 19 K (details see Supplementary Section S2). A schematic of the geometry is shown in Fig. 1b. The K_3_C_60_ films were connected to three pairs of photo-conductive switches, consisting of amorphous silicon patches fabricated along the signal lines of coplanar waveguides. When irradiated with 250 fs-long, 515 nm-wavelength laser pulses, the biased photo-conductive switches become transiently conductive, and launch ultrafast electrical pulses. Their duration is determined by the lifetime of the photo-carriers in the silicon switches (~300 fs, see Fig. S8) and by the bandwidth of the waveguide (~1 THz, see Fig. S11). In all the measurements reported here, the two leftmost switches were excited simultaneously, to launch quasi-transverse electromagnetic (TEM) pulses along the signal line (details see Supplementary Section S3.4).

Before interaction with the K_3_C_60_ thin film, the electrical pulses were sampled in a second pair of switches (see Fig. 1b), providing a reference voltage transient V_1_(t). Unlike for the first pair of switches, this second pair was left unbiased, yielding a measurable current only when both the electrical pulse and a femtosecond optical gate were superimposed on the switch. By scanning the mutual delay (denoted here as sampling time *t*) between the electrical pulse and the optical gating pulse, V_1_(t) could be measured (see representative transient in Fig. 1b). The pulse transmitted after interaction with the K_3_C_60_ sample was measured in the same way at a third pair of photo-conductive switches, yielding a second time-resolved trace V_2_(t), displayed in Fig. 1b.

A few characteristics are immediately apparent by inspecting the V_2_(t) transients transmitted by the sample at equilibrium. For temperatures *T* > *T*_c_, for which the sample was resistive, the voltage pulse was attenuated. For the low-temperature superconductor (*T* < *T*_c_), the transmitted V_2_(t) pulse was not attenuated, but was broadened in time. This effect is well understood by noting that superconducting K_3_C_60_ acts as lumped inductor, with no dissipation but with frequency dispersion (Ẑ = jωL). Note also that for *T* < *T*_c_ the inductive response of the film causes a ~500 fs group delay relative to the resistive response, as expected.

**Fig. 2:**
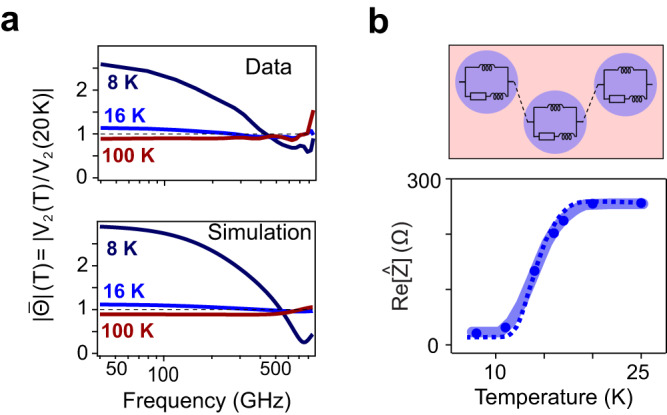
Probing the equilibrium superconducting transition with ultrafast transport. **a** Upper: normalized transmittance ${\left|\overline \varTheta \right|}$ measured at 8 K, 16 K, and 100 K. Lower: corresponding simulation using the effective circuit model shown in **b**. The black dashed lines indicate unity. **b** Upper: schematic of circuit model used in **a**. Blue circles indicate superconducting grains and red area indicates the resistive weak coupling between grains. Lower: real part of the impedance Re[Ẑ] at 50 GHz (blue dots) as a function of temperature. The resistance versus temperature curve measured with DC transport on the same device is plotted as blue dashed line.

The same data is presented as a frequency-resolved transmittance $\varTheta \left(\omega,\,T\right)={V}_{2}(\omega,\,T)/{V}_{1}(\omega,\,T)$ in Fig. 2a, plotted as $\bar{\varTheta }\left(\omega,\,T\right)=\frac{\varTheta \left(\omega,T\right)}{\varTheta \left(\omega,20{K}\right)}=\frac{{V}_{2}\left(\omega,T\right)}{{V}_{2}\left(\omega,20{K}\right)}$ after normalization by the transmittance measured immediately above *T*_c_. Note that the modulus of $\bar{\varTheta }\left(\omega,T\right)$ was larger than 1 for all temperatures *T* < *T*_c_ and smaller than 1 for all temperatures *T* > *T*_c_ (more temperatures see Fig. S29). The dip in normalized transmittance near 700 GHz, observed only in the superconducting state, reflects an LC resonance formed by the inductance of the superconductor L_s_ and the capacitance C_c_ between the contacts on the K_3_C_60_ film, which is confirmed by consistency between the data and simulation (See Supplementary Section S6.2).

To compare these ultrafast measurements with the DC response, the complex impedance $\hat{Z}$ was extracted from the complex valued $\bar{\varTheta }\left(\omega,\,T\right)=\frac{2*{Z}_{w}+\hat{Z}(\omega,20K)}{2*{Z}_{w}+\hat{Z}(\omega,T)}$. In this expression, ${Z}_{w}$ denotes the wave impedance of the coplanar waveguide (see Fig. S12). The real part of $\hat{Z}(\omega,\,T)$ is displayed in Fig. 2b (blue dots), together with the two-point resistance measurement at DC (blue dashed line), yielding a qualitatively similar scaling.

Since these films are polycrystalline (see AFM image in Fig. S14), the equilibrium transition from the metallic state at *T* > *T*_c_ to a purely inductive superconductor (*T* < 0.6 *T*_c_) is broadened. This is understood by considering a granular superconductor^33–40^ composed of a set of grains undergoing a sharp superconducting transition at *T*_c_, coupled to one another by weak links (Fig. 2b). Unlike the superconducting grains, these weak links exhibit finite resistivity down to appreciably lower temperature than *T*_c_ due to thermally-activated phase slips, causing a gradual reduction in the overall resistance (see Supplementary Section S6.3).

**Fig. 3:**
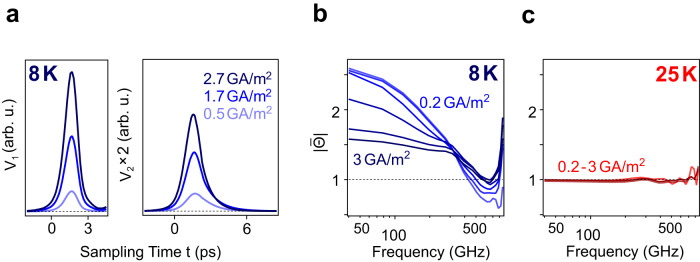
Nonlinear ultrafast transport response of the equilibrium superconducting state. **a** Incoming and transmitted electrical pulses with different peak current density. The peak current density of the transmitted pulses is labelled. **b**, **c** Current- and frequency-dependent normalized transmittance $\left|\bar{\varTheta }\right|$ at 8 K (**b**) and 25 K (**c**). The peak current density of the transmitted pulses is labelled. The black dashed lines indicate unity.

The nonlinear electrical response of the sample, measured with the same ultrafast device of Fig. 1b, confirms the picture of a granular superconductor qualitatively highlighted above. In these measurements, the peak current density of the probe pulses was controlled by changing the bias voltage of the leftmost launching switches. Figure 3a shows representative V_1_(t) and V_2_(t) measurements at *T* = 8 K (<<*T*_c_).

The nonlinear transmittances of the superconducting state are shown in Fig. 3b, contrasted with measurements in the metallic state at 25 K (Fig. 3c). As expected, the transmittance of the equilibrium superconducting state reduces with increasing peak current density, whilst the equilibrium metallic state at 25 K exhibits no dependence on the current density.

All of these observations are explained by considering that the onset of resistive response is assigned to a combination of thermally generated and current-induced phase slips at the weak links between superconducting grains. At the lowest temperatures, where the superconductivity is well-formed and the DC resistivity vanishes (e.g., *T* = 8 K), these phase slips are infrequent, and linear charge transport is dominated by inter-grain coherent tunneling. When, at these lowest temperatures, the current density reaches *J*_c_, phase slips are induced electrically^35–38,41^, leading to a finite resistance that grows with bias current. The resistance growth is less steep for picosecond current pulses than for DC current bias, because the phase slips are induced only over the duration of the electrical pulse, and are less efficiently generated for short pulse durations (see Supplementary Section S5.1).

**Fig. 4:**
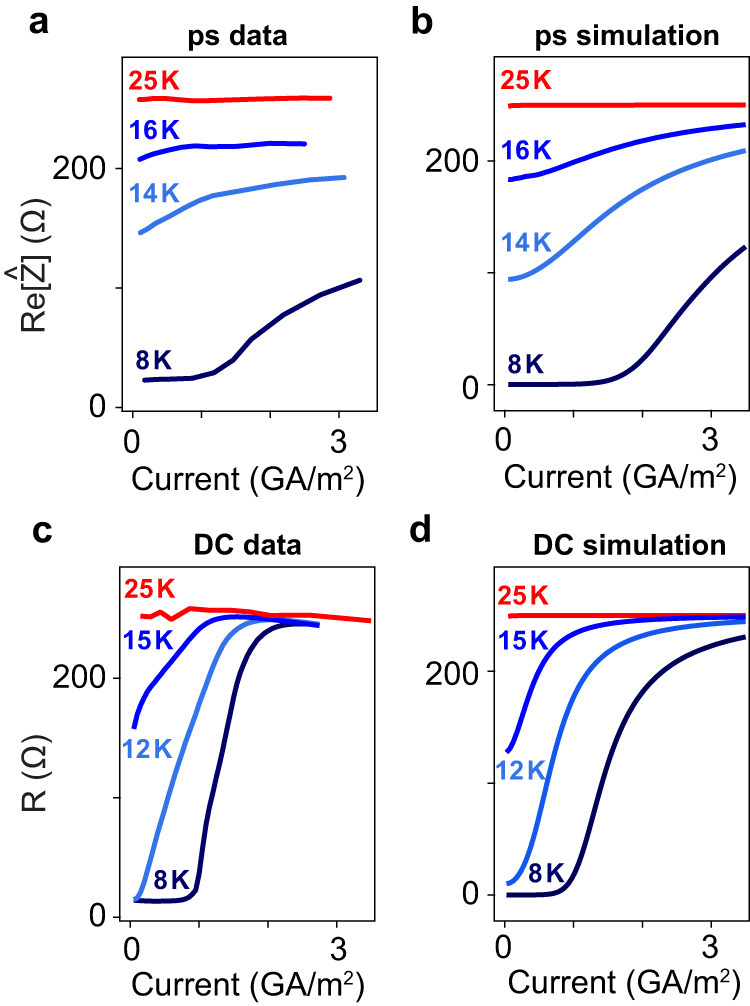
Critical current of the equilibrium superconducting state in the picosecond and DC time scale. **a**, **b** Ultrafast transport measurements (**a**) and simulations (**b**) of Re[Ẑ] at 50 GHz versus peak current density at 8, 14, 16, and 25 K. **c**, **d** DC transport measurements (**c**) and simulations (**d**) of resistance versus current density at 8, 12, 15, and 25 K.

At intermediate temperatures where the superconducting grains are weakly coupled, phase slips are activated thermally already in the absence of a strong current, causing a finite small-signal resistance^35–38^. With increasing current, the resistance is increased by induced phase slips. This description is also verified by the scaling behavior $\triangle V \sim {(I-{I}_{c})}^{\alpha }$ in DC transport measurements, where $\alpha$ changes from 1 to 3 when the temperature is reduced from 16 to 8 K (see Fig. S17)^38,41–43^. To compare DC and picosecond transport measurements, we simulated the measured data reported in Fig. 4a, c for both picosecond and DC currents at different temperatures. The simulation was conducted by considering the equation of motion for Josephson junctions, similar to what was described in ref. ^33,34^. The results are shown in Fig. 4b (picosecond simulation) and Fig. 4d (DC simulation), which reproduce the key features of the experiments (See Supplementary Section S6.3).

We next turn to the non-equilibrium photo-induced state, in search of transient signatures of superconductivity similar to those reported in Figs. 3 and 4. The optical-pump-electrical-probe delay *τ* (different from the sampling time *t* of Figs. 2 and 3), was scanned by using a mechanical delay line for the pump beam. The data was obtained when the K_3_C_60_ film was held at *T* = 25 K and was excited with mid-IR (*λ* = 7 µm) pulses at fluences up to 4 mJ/cm^2^.

**Fig. 5:**
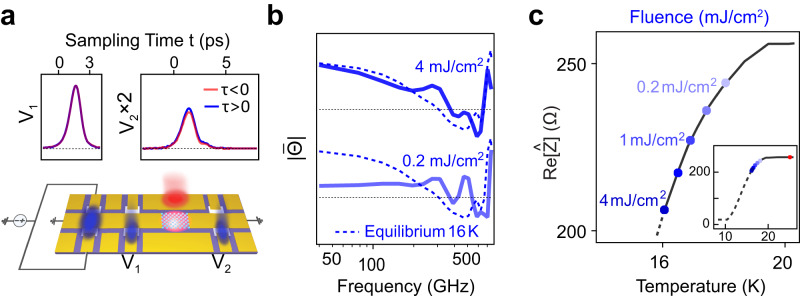
Reduction of the impedance in the photo-induced state. **a** Lower: schematic of the ultrafast transport measurement of the photo-induced state in K_3_C_60_. The red beam on the K_3_C_60_ thin film indicates the mid-IR excitation. Upper: incoming pulse V_1_(t) and transmitted pulse V_2_(t) at *τ* < 0 and *τ* > 0. *τ* is the time delay between electronic pulse and mid-IR pulse, defined as $\tau={{t}_{e-{pulse}}-t}_{{mid}-{IR}}$ where ${t}_{e-{pulse}}$ and ${t}_{{mid}-{IR}}$ are the arriving times of the electronic pulse and the mid-IR excitation, respectively. **b** Normalized transmittances for pump fluences 0.2 and 4 mJ/cm^2^ at 25 K. 4 mJ/cm^2^ data is offset for clarity. The transmittance at equilibrium 16 K is plotted as blue dashed lines for comparison. The two black dashed lines indicate unity. **c** Dependence of Re[Ẑ] on pump fluence in the photo-induced state (upper axis). Dots from right to left (lighter to darker blue) are measurements at 0.2, 0.5, 1, 2, and 4 mJ/cm^2^. Re[Ẑ] versus temperature (lower axis) in the equilibrium state is shown by a black line for comparison. Here pump-probe measurements are taken with mid-IR pump at *T* = 25 K and at *τ* = 5 ps. The peak current density used in **b** and **c** is ~0.1 GA/m^2^. Inset: Reduction of Re[Ẑ] of the photo-induced state compared with the equilibrium superconducting transition. The red dot indicates the position of the unpumped state at 25 K.

Similar to what was observed when cooling the sample below *T*_c_, photo-excitation caused the transmitted pulse V_2_(t) to increase in amplitude, indicating a reduction in resistance. The normalized transmittances at two pump fluences 0.2 and 4 mJ/cm^2^ are presented in Fig. 5b (more fluence-dependent data can be found in Supplementary Section 5.2). The key result is that the transmittance of the photo-excited K_3_C_60_ film saturates to the level of the granular 16 K equilibrium response (*T* = 0.8 *T*_c_), displayed for comparison in the plot (dashed lines). The calculated Re[Ẑ] at different pump fluences is shown in Fig. 5c, along with the equilibrium superconducting transition curve for comparison.

**Fig. 6:**
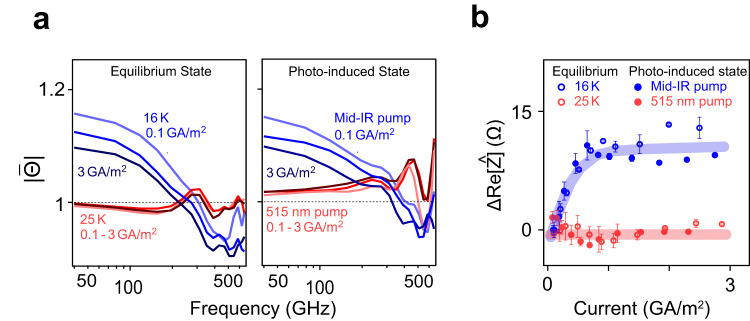
Critical current of the photo-induced state. **a** Left: current dependence of the transmittance in equilibrium at 16 K (blue) and at 25 K (red). Right: current dependence of the transmittance of the photo-induced state with mid-IR excitation with a fluence 4 mJ/cm^2^ (blue) and 515 nm excitation with a fluence 4 mJ/cm^2^ (red). **b** Change of the real part of the impedance Re[Ẑ] with increasing current in equilibrium at 16 K (blue circle), at 25 K (red circle), and for the photo-induced state with mid-IR excitation (blue dots) and 515 nm excitation (red dots). The error bars shown are defined as standard errors.

The same conclusion was drawn when probing the transient nonlinear transport properties. Figure 6a displays the normalized transmittance of the equilibrium state (left) and the photo-induced state (right) at different peak current densities. Just as in the transient linear impedance, the nonlinear transport properties of the photo-induced state showed the same behavior as that observed in equilibrium at 16 K.

The observation of a photo-induced state with nonlinear I–V characteristics is uniquely indicative of non-equilibrium superconductivity. In fact, when the same film was excited with visible light pulses (515 nm), for which no photo-induced superconductivity is expected^7^, this nonlinearity disappeared. This comparison is summarized in Fig. 6b, where the corresponding changes of Re[Ẑ] are plotted as a function of peak current density. In this panel, the nonlinear electrical transport of the photo-induced superconducting state is compared with the equilibrium granular superconductor at *T* = 16 K, and contrasted with the response of the equilibrium metal and of that excited in the visible, for which no nonlinearity was observed.

In seeking to interpret the nature of the photo-induced state, we propose that the effect of the mid-infrared pump is to turn the grains into a transient superconducting state, whilst the weak links connecting these regions are left in an excited state. This excitation at the weak links may be a result of energy deposited in the films, or even of a fast quench that drives the order parameter phase to different values in neighboring grains. Hence, the observation that even at the highest fluences we could not induce properties observed in equilibrium below *T* = 16 K (0.8 *T*_c_) may be a result of the polycrystalline nature of the sample and of weak links that heat upon photo-excitation. It is not clear that the pressed powders used in the previously reported optical studies would exhibit the same type of transient granular superconductivity documented here. Systematic work is needed for different film configurations and thicknesses, as well as further optimization of the growth process.

The above interpretation appears more likely than one in which the finite residual resistance of the photo-induced state is attributed to a fraction of the sample becoming superconducting after excitation, leaving another fraction metallic. This is predicated on the change in resistance versus temperature at different bias currents (See Fig. S16), which does not exhibit double transition features^44,45^ and indicates the stoichiometric homogeneity of the thin-film sample. Secondly, the fact that the sample is pumped by an optical beam with a size similar to the sample size (FWHM of pump beam ~21 µm and sample dimensions 20 × 20 µm²), makes an inhomogeneous photo-induced state less convincing at this stage.

In summary, we have developed a new experimental platform to probe ultrafast nonlinear transport in doped fulleride thin films. We report unique signatures of photo-induced superconductivity as nonlinear I-V characteristics in the transient state. In these devices, which are based on polycrystalline films, the photo-induced state does not acquire the transport properties of the homogeneous superconductor and rather saturates into a non-equilibrium state with both linear and nonlinear transport properties similar to those observed in equilibrium for *T* ~ 0.8 T_c_. This is possibly a result of the polycrystalline geometry of the samples, although we cannot exclude that under all circumstances, fast quenches may leave a photo-induced state with a high density of vortex excitations and other topological defects. Future work could be extended to different superconductors, whether by using new growth protocols or by exfoliating layered materials such as Bi-based cuprates and van der Waals-based superconductors. These studies will also make accessible a wider variety of non-equilibrium phenomena in quantum materials, including topological states, sliding charge and spin density waves as well as domain wall propagation. Finally, the work reported here represents an important step towards the integration of quantum materials into ultrafast electrical devices, which may lead to high-bit-rate applications based on the functionalities of driven quantum materials.

## Methods

### Device preparation

The photo-conductive switches and coplanar waveguide were fabricated on a sapphire substrate using laser lithography and electron-beam evaporation. A bilayer photoresist mask was used for the lithography, for which MicroChem LOR-7B served as an undercut layer and micro resist ma-P 1205 served as the light-sensitive top layer. After the first developing process, a 200 nm layer of Si was evaporated for the photo-conductive switches. After the second developing process, 10 nm Ti/280 nm Au was evaporated to form the coplanar waveguide structure.

Afterwards, a shadow mask with a 20 µm × 20 µm square hole in the center was aligned to the middle of the device under an optical microscope using a micro-manipulator. The whole device was then transferred to an MBE chamber and was degassed at 300 °C for 12 hours prior to growth. The C_60_ molecules were deposited on the device from an effusion cell source with the device kept at 200 °C and the C_60_ source at 380 °C. The deposition rate was ~1 nm/min. The thickness of the C_60_ thin film was ~100 nm. To improve the electrical contact between the C_60_ thin film and the signal line of the waveguide, 10 nm Ti/350 nm Au was deposited additionally onto the contact area with another shadow mask.

The C_60_ film was doped with potassium with the device kept at 200 °C and the potassium source at 100 °C. The resistance of the film was monitored with an ohmmeter through electrical feedthroughs. The doping process was stopped when the sample’s resistance reached a minimum. After doping, the device was transferred to a glovebox using a high-vacuum suitcase. The device is then attached to a home-made printed circuit board (PCB) and sealed with a diamond window with an indium gasket. The electrical pads on the device were connected to the PCB via electrical feedthroughs fabricated with low vapor pressure epoxy Torr Seal.

### Optical setup

The optical part of the experimental setup is based on a Pharos Laser, which provides optical pulses with pulse energy 400 µJ and duration 250 fs, with a wavelength centered at 1030 nm and a repetition rate of 50 kHz. The 7 μm optical pulses were generated with an optical parametric amplifier (OPA) utilizing silver thiogallate (AGS) crystals. The 515 nm optical pulses were generated by second harmonic generation with a Beta Barium Borate (BBO) crystal. The 7 µm pulses and the 515 nm pulses were directed into a Janis ST-500 optical cryostat through an imaging system based on two pellicle beam splitters and a reflective objective to focus them onto the device.

### Data collection

During all measurements, the electrical signals from the photo-conductive switches were amplified by an in-house custom-built trans-impedance amplifier via electrical feedthroughs from the cryostat and then measured using a lock-in amplifier. The 515 nm light used to launch the electrical pulses was chopped at ~1 kHz, which was used as the reference frequency of the lock-in amplifier. Optical delay lines were used to scan the mutual time delay between the launching and the sampling 515 nm pulses, and to adjust the delay of the 7 µm pulses relative to both 515 nm pulses. The data were recorded using custom LabVIEW software.

### Supplementary information

Supplementary Information

Peer Review File

### Source data

Source Data

## Data Availability

Source data for Figs. 1–6 are provided in online version of this paper. Additional data are available from the corresponding author upon request. Source data are provided with this paper.
