# Supplementary material for: Superconducting nonlinear transport in optically driven high-temperature K3C60
Source: Nat Commun. 2023 Nov 9;14:7233. doi: 10.1038/s41467-023-42989-7 (PMC10636163; doi:10.1038/s41467-023-42989-7)
Supplement: Supplementary file 1 — Supplementary Information [file 41467_2023_42989_MOESM1_ESM.pdf]

Supplementary information for

Superconducting nonlinear transport in optically driven high temperature K_3C_{60}

E. Wang[1], J. D. Adelinia[1], M. Chavez-Cervantes[1], T. Matsuyama[1], M. Fechner[1], M. Buzzi[1], G. Meier[1], A. Cavalleri[1,2]

[1] *Max Planck Institute for the Structure and Dynamics of Matter, Hamburg, Germany*

[2] *Department of Physics, Clarendon Laboratory, University of Oxford, United Kingdom*

Contents

S1. Experimental setup 2

S2. Device fabrication process 4

S3. Device and sample characterization 6

S3.1: Characterization of the launched electrical pulse 6

S3.2: Lifetime of photo-carriers in the photo-conductive switches 7

S3.3: Calibration of photo-conductive switches 8

S3.4: Launching of an ultrashort quasi-TEM electrical pulse 9

S3.5: Characterization of the coplanar waveguide: Damping and impedance 9

S3.6: Calibration of peak current value of ultrashort electrical pulse 11

S3.7: Characterization of the K_3C_{60} thin film 11

S4. Data analysis 14

S4.1: Subtraction of the reflected signal 14

S4.2: Double-exponential fitting of data 16

S5. More data 20

S5.1: Measurements with a nanosecond pulse generator 20

S5.2: Ultrafast transport measurements 22

S5.2.1: Photo-induced state below and above T_c 22

S5.2.2: Fluence dependence 23

S6. Simulation 24

S6.1: Time-domain simulation 24

S6.2: Frequency-domain calculation 25

S6.3: Critical current simulation 26

S1. Experimental setup

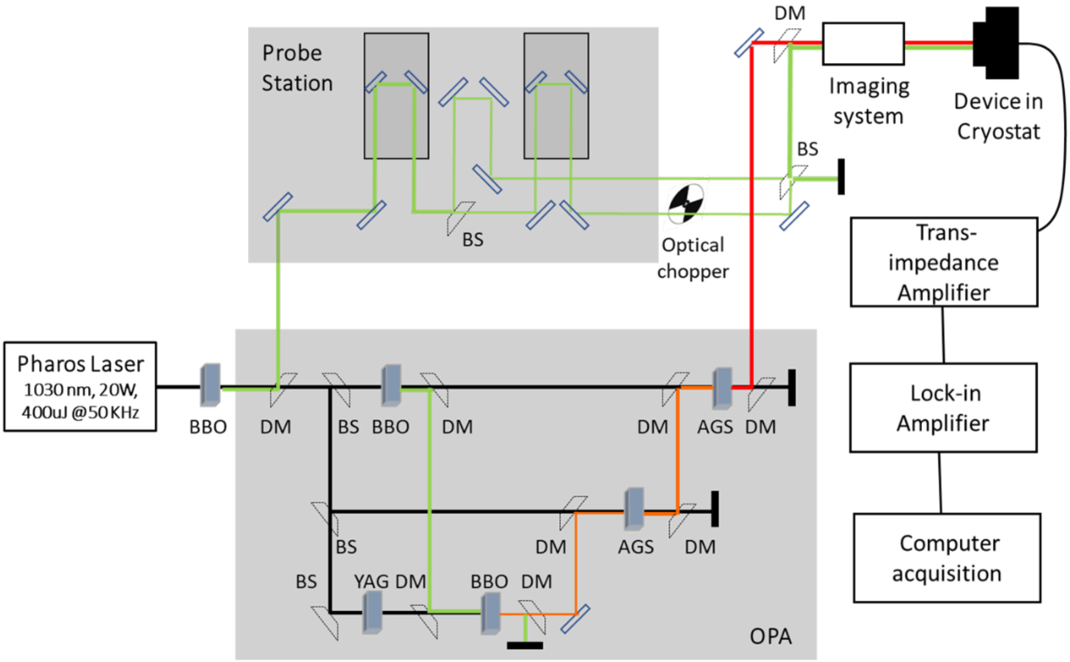

Fig. S 1| Schematics of the experimental setup. Abbreviations of objects: BBO-Beta Barium Borate crystal; DM-Dichroic Mirror; BS-Beam Splitter; YAG-Yttrium Aluminum Garnet crystal; AGS-Silver Thiogallate crystal; OPA-Optical Parametric Amplifier. The wavelength of optical pluses is represented by color: 515 nm (green), 1030 nm (black), 1200 nm (orange) and 7 µm (red).

The optical part of the experimental setup is based on a Pharos Laser, which provides optical pulses with pulse energy 400 µJ and duration 250 fs, with a wavelength centered at 1030 nm and a repetition rate of 50 kHz (Fig. S 1). The output pulses were first sent through a 0.1 mm thick Beta Barium Borate (BBO) crystal to generate visible pulses with wavelength 515 nm. The 515 nm pulses and the leftover 1030 nm pulses were separated using a dichroic mirror (DM). The 515 nm pulses were used to excite photo-conductive switches on the sample device. The leftover 1030 nm pulses were used to generate mid-Infrared pulses with an optical parameter amplifier (OPA) utilizing silver thiogallate crystals based on the design of reference [1]. The mid-IR pulses (7 µm) from the OPA and the 515 nm pulses were directed into a Janis ST-500 optical cryostat through an imaging system based on a pellicle beam splitter and a reflective objective to focus them onto the device. The spectrum of the mid-IR pulses was characterized by Fourier Transform InfraRed interferometry (Fig. S 2) and the beam diameter in the focal plane was measured using a typical knife-edge method (Fig. S 3).

During all measurements, the electrical signals from the photo-conductive switches were amplified by an in-house custom-built transimpedance amplifier via electrical feedthroughs from the cryostat and then measured using a lock-in amplifier. The 515 nm light used to

launch the electrical pulses was chopped at approximately 1kHz, as indicated in Fig. S 1, and this frequency was used as the reference frequency of the lock-in amplifier. Optical delay lines were used to scan the mutual time delay between the launching and the sampling 515 nm pulses, and to adjust the delay of the 7 µm pulses relative to both 515 nm pulses. The data was recorded using custom LabVIEW software.

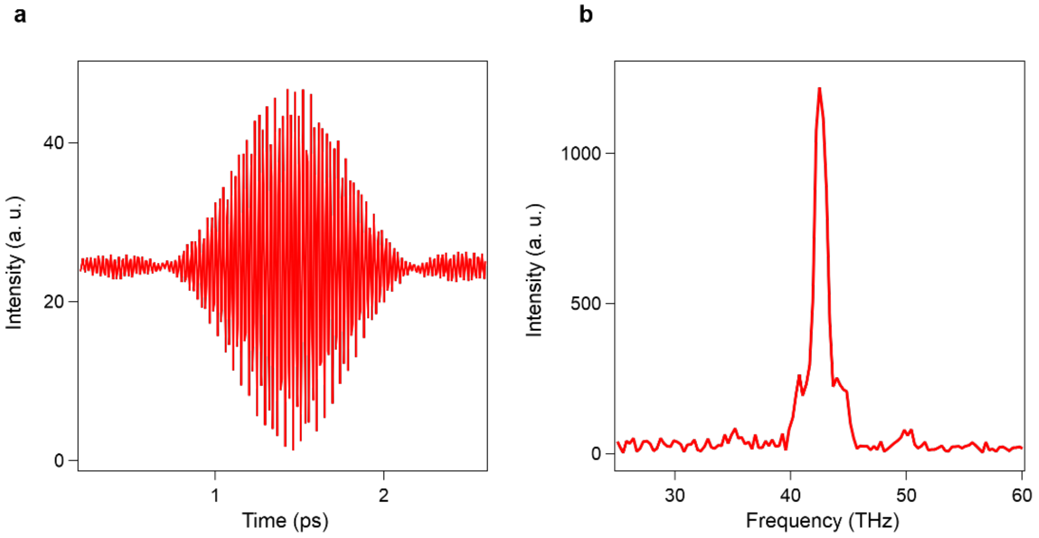

Fig. S 2| Fourier Transform InfraRed (FT-IR) spectrum of the mid-IR. (a) Time trace of the interference signal from the Michelson interferometer. (b) Fast Fourier transform of the time trace in (a).

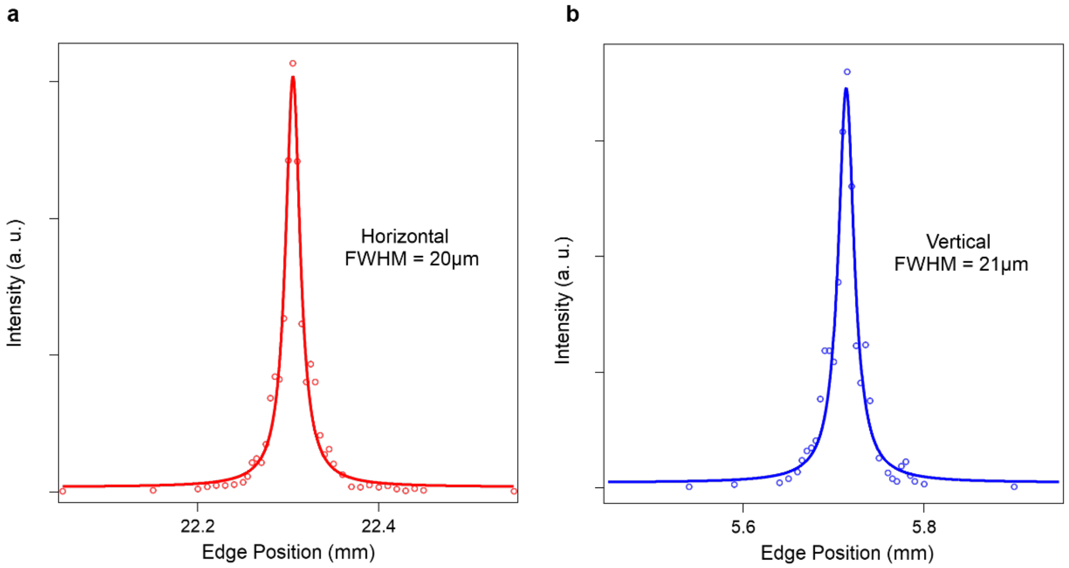

Fig. S 3| Horizontal and vertical beam size of the mid-IR pulses at the focal plane of the imaging system, measured using the knife-edge method.

S2. Device fabrication process

Prior to K_3C_{60} thin film growth, the photo-conductive switches and coplanar waveguide were fabricated on a sapphire substrate using laser lithography and electron-beam evaporation. This was done in two steps – first the switches, then the waveguide. For both steps, a bilayer photoresist mask was used for the lithography, for which MicroChem LOR-7B served as an undercut layer and micro resist ma-P 1205 served as the light-sensitive top layer. After developing the structure, a 200 nm layer of silicon was evaporated for the photo-conductive switches (Fig. S 4a). In the second step, 10 nm of titanium immediately followed by 280 nm of gold was evaporated to form the coplanar waveguide structure (Fig. S 4b).

Afterwards, a shadow mask with a 20 µm x 20 µm square hole in the center was aligned to the middle of the device under an optical microscope using a micro-manipulator (Fig. S 4c). The whole device was then transferred to a molecular beam epitaxy (MBE) chamber and was degassed at 300 °C for 12 hours prior to growth. The C_{60} molecules were deposited on the device from an effusion cell source with the device kept at 200 °C and the C_{60} source at 380 °C. The deposition rate was ~ 1 nm/min. The thickness of the C_{60} thin film was ~100 nm. During growth, the temperature of the device was monitored using a pyrometer.

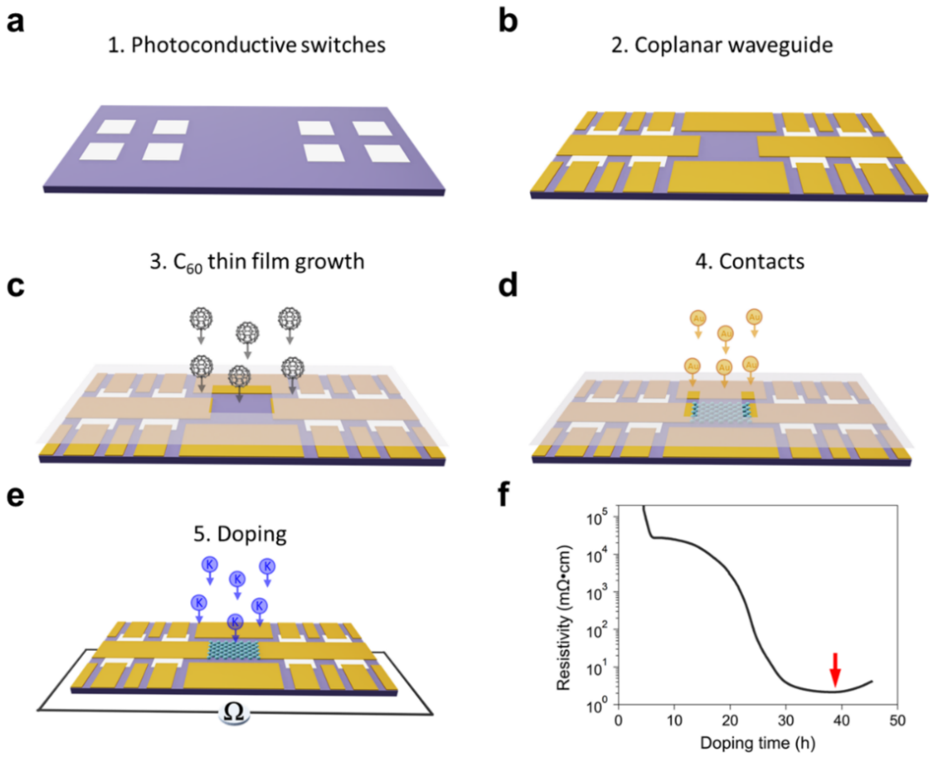

Fig. S 4| Schematics of device fabrication steps (a) to (e). (f) Resistivity profile of the doping process.

To improve the electrical contact between the C_{60} thin film and the signal line of the waveguide, 10 nm Ti/350 nm Au were deposited additionally onto the contact area (Fig. S 4d).

To obtain a K_3C_{60} film, potassium was deposited on the C_{60} thin film from an effusion cell source with the device kept at 200 °C and the potassium source at 100 °C. To precisely control the stoichiometry, the device was connected to an ohmmeter via electrical feedthroughs from the chamber and the resistance of film was monitored in-situ during the doping process (Fig. S 4e). The resistance decreases upon initial doping and subsequently reaches a minimum (indicated by the red arrow in Fig. S 4f), which corresponds to the formation of K_3C_{60} as reported in previous studies [2, 3]. A superconducting transition with T_c ~ 19 K as shown in the main text and below further confirms the correct stoichiometry. In order to achieve homogeneous doping, a dope-anneal cycle consisting of 1 hour of doping followed by a 6-hour anneal was used to allow enough time for potassium to diffuse inside the film.

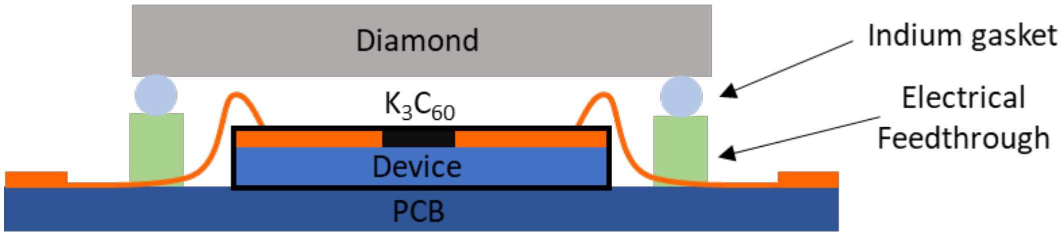

Fig. S 5| Schematics of the device after sealing.

After doping, the device was transferred to a glovebox using a high-vacuum suitcase to avoid oxidation. In the glovebox, the device was attached to a home-made printed circuit board (PCB), and sealed with a diamond window together with an indium gasket (Fig. S 5). The electrical pads on the device were connected to the PCB via electrical feedthroughs fabricated with low vapor pressure epoxy Torr Seal. After sealing, the device was transferred into the optical cryostat for measurements. During the measurements, the optical pulses were incident on the device through the top diamond window.

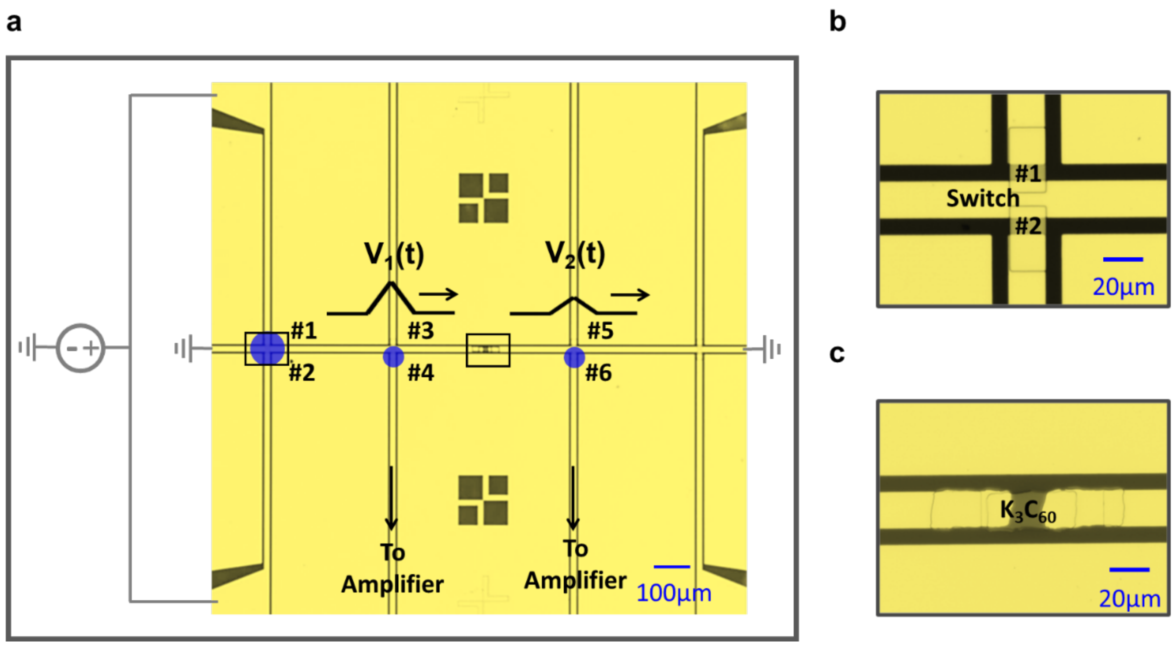

Fig. S 6| Optical microscope image of the device. #1-#6 denote the three pairs of photo-conductive switches. $V_1(t)$ and $V_2(T)$ denote the reference and transmitted electrical pulse. (b, c) Zoom-in images of a pair of photo-conductive switches (b) and K_3C_{60} thin film (c) as indicated by black boxes in (a).

A microscope image of the device is shown in Fig. S 6. The three pairs of photo-conductive switches are labelled #1 to #6. The zoom-in images of switches and K_3C_{60} thin film are shown in Fig. S 6b and c. During the measurements, switches #1 and #2 were biased simultaneously and were illuminated together with 515 nm pulses (with fluence ~ 4 mJ/cm^2), to launch the electrical pulse $V_1(t)$. $V_1(t)$ was sampled at switch #4 with another 515 nm pulse (~4 mJ/cm^2). After measuring $V_1(t)$, the transmitted electrical pulse $V_2(t)$ was sampled by illuminating switch #6 instead of switch #4. The corresponding electrical signals from both detection switches were collected using transimpedance and lock-in amplifiers.

S3. Device and sample characterization

S3.1: Characterization of the launched electrical pulse

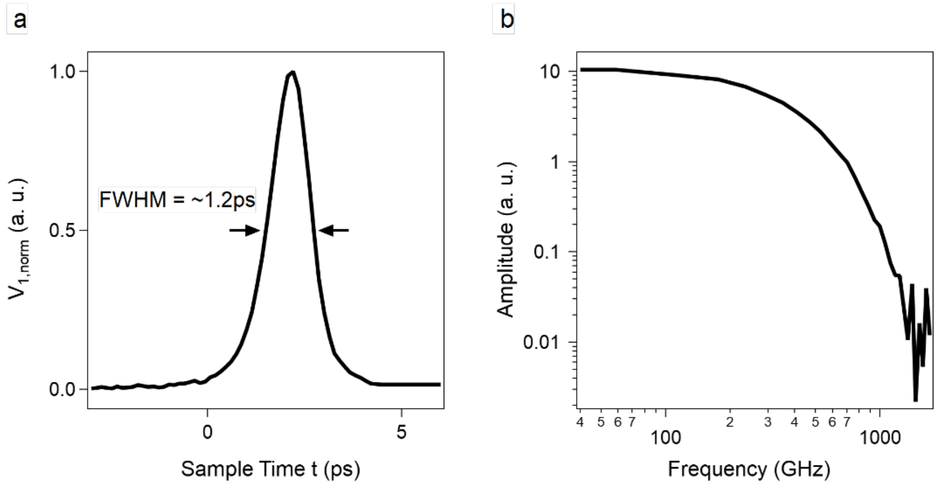

Fig. S 7|Temporal width and spectrum of the launched electrical pulse. (a) Time trace of the launched electrical pulse, which is the reference pulse $V_1(t)$ in experiment. The shown curve is normalized to unity. (b) Fast Fourier transform of the time trace in (a) to show the spectrum in frequency domain.

The time trace of the electrical pulse launched from photo-conductive switches #1 and #2, is shown in Fig. S 7a. The full width at half maximum (FWHM) is ~1.2 ps and the spectral weight of pulse is very weak above 1 THz (Fig. S 7b). The limiting factors for the pulse width are the lifetime of photo-carriers in the switches and the bandwidth of coplanar waveguide used in the experiment. These will be discussed in the following sections S3.2 and S3.5.

S3.2: Lifetime of photo-carriers in the photo-conductive switches

The lifetime of the photo-carriers in the photo-conductive switches was determined by measuring the auto-correlation curves of the switch response functions (proportional to the time profile of the photo-carrier population). During the measurement, we illuminated switches #3 and #4 with two small-diameter 515 nm pulses which each only covers one switch. Meanwhile, one switch was biased with a voltage and the other one remained unbiased (Fig. S 8a). By changing the mutual delay between the two 515 nm pulses (denoted as sampling time t), a time profile was measured which was the auto-correlation of the switch response functions (Fig. S 8c). The measured time profiles are simulated by assuming the response function has a rising time the same as the pulse duration of the 515 nm pulse (~ 250 fs) and a decay time the same as the photo-carrier lifetime τ_e (Fig. S 8b). Fig. S 8c shows the comparison between measurements and different simulation results with various τ_e values. The photo-carrier lifetime τ_e is estimated to be between 250 – 300 fs. This result was observed to be independent of temperature between 8 and 50 K.

The photo-carrier lifetime in our experiment is shorter than that observed in previous studies [4]. This is possibly caused by the annealing process before C_{60} deposition, which could cause the migration of titanium or gold atoms into the silicon patches and increase the scattering rate.

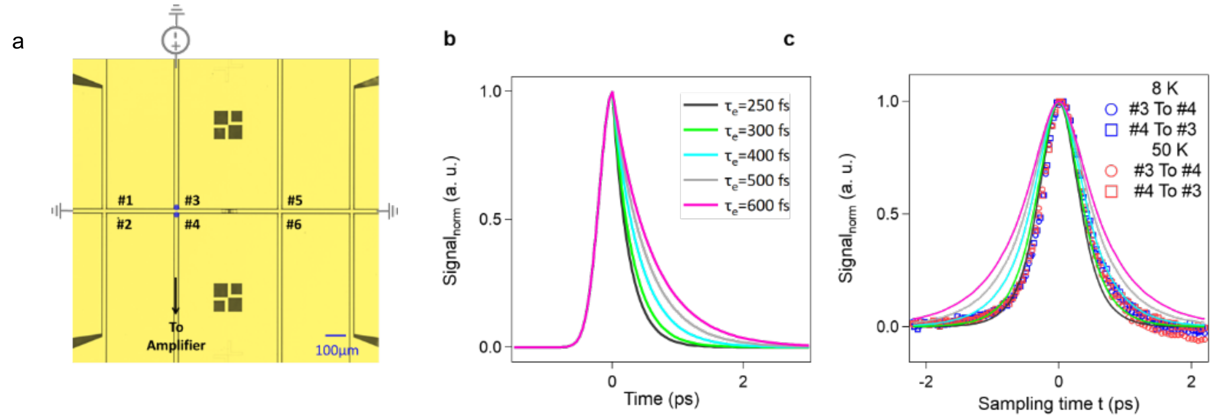

Fig. S 8| Lifetime of the photo-carriers in the photo-conductive switches. (a) Setup of measurement. (b) Response function of the photo-conductive switch, with a rising time of ~ 250 fs, for various photo-carrier lifetimes τ_e. (c) Comparison of the auto-correlation of the response function and the measurements by exciting switches #3 and #4. The geometry in (a) corresponding to measurement #3 to #4, in which switch #3 is biased and #4 is connected to transimpedance amplifier. In measurement #4 to #3, this is reversed.

S3.3: Calibration of photo-conductive switches

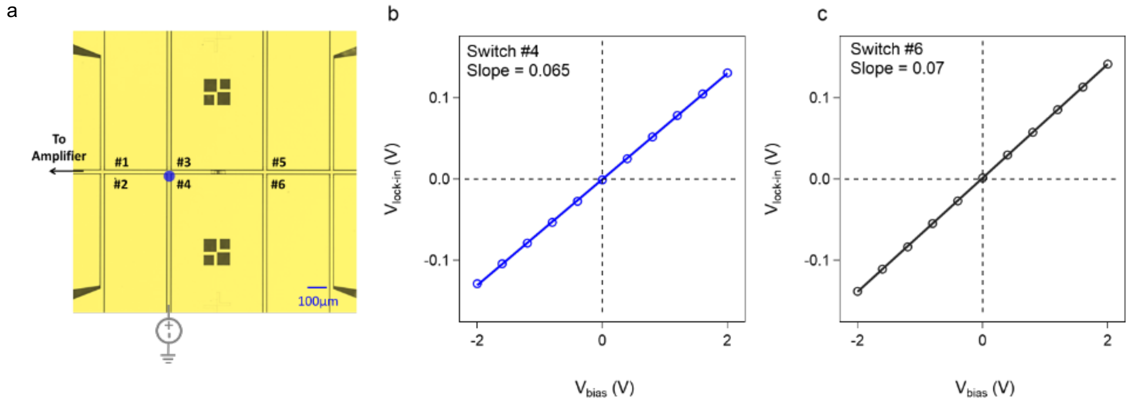

Fig. S 9| Calibration of the photo-conductive switches. (a) Setup for calibration process for switch #4. The switch being calibrated was biased with a voltage and illuminated with 515 nm pulses. (b, c) The signal measured with the lock-in amplifier versus the bias voltage. The slope was calculated for each switch and used as the normalization factor individually.

During the experiments, it was difficult to ensure that the 515 nm pulses used to illuminate all switches were identical, which means that during the measurements each switch had a slightly different sensitivity. To calibrate this difference, before each measurement, the switch that was used to probe the electrical pulse was calibrated independently. The switch being calibrated was biased with a voltage and illuminated with 515 nm pulses. The generated electrical signal was collected at one end of the signal line (the other end was left open) through the combination of a transimpedance amplifier and a lock-in amplifier (Fig. S 9a). The dependence of the signal on the bias voltage is shown in Fig. S 9(b, c) for the two switches which were used to sample the reference pulse $V_1(t)$ and the transmitted pulse $V_2(t)$, respectively. The sensitivity of the switch is directly represented by the slope of the

curve which was used as a normalization factor to normalize the data in our experiment. For each measurement in the experiment, the sensitivity of the switch which was used to sample the electrical pulse was calibrated beforehand.

S3.4: Launching of an ultrashort quasi-TEM electrical pulse

In the experiment, it is important that the launched ultrashort electrical pulse has a single quasi-TEM mode, as different excitation modes have different impedance, which would make the analysis difficult. In our case with the coplanar waveguide, switches #1 and #2 (Fig. S 6) were biased simultaneously and symmetrically using a voltage source, and were illuminated uniformly. In this way, the electrical pulse was launched in a symmetrical way as even mode (meaning the electrical field is symmetrical about the signal line), which is the quasi-TEM mode in the coplanar waveguide. To confirm, we measured the electrical pulse with two switches #3 and #4, which are on the upper and lower side at the same position of the signal line. The measurements yielded almost identical signals as shown in Fig. S 10, indicating that we launch an even mode into the waveguide with this technique.

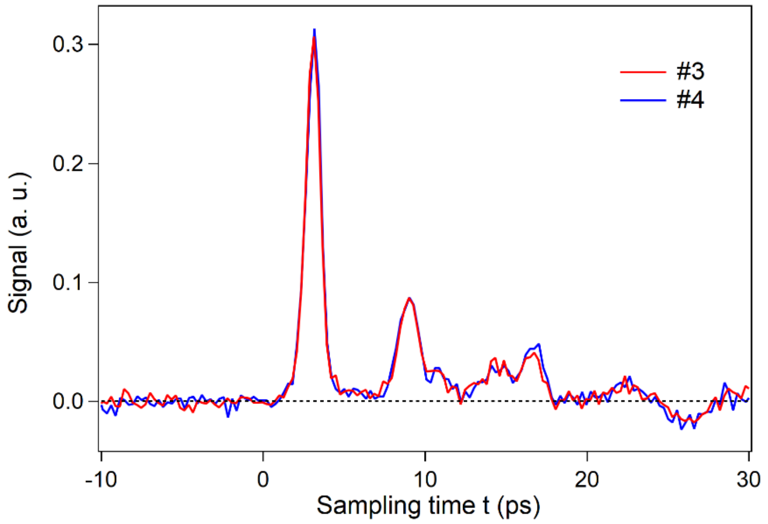

Fig. S 10| Characterization of the launched electrical pulse. The launched electrical pulse was sampled using switches #3 and #4. The signals are proportional to the electrical field on the two sides of signal line.

S3.5: Characterization of the coplanar waveguide: Damping and impedance

Other than the photo-carrier lifetime, another limiting factor to the temporal width of generated electrical pulse is the damping caused by the coplanar waveguide used in the experiment. The frequency-dependent transmittance of the quasi-TEM pulse for a 200 µm long waveguide, extracted from a finite-element time-domain simulation in CST studio suite, is shown in Fig. S 11. The frequency component above 1 THz is strongly damped, with the -6

dB point at approximately 1 THz. This is consistent with the electrical pulse width of ~ 1 ps observed in the experiment.

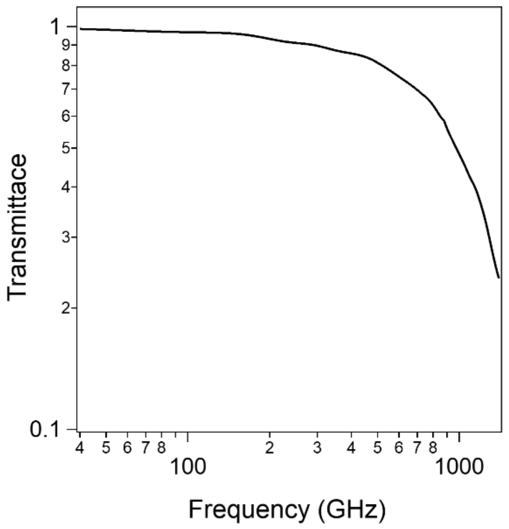

Fig. S 11| Frequency domain finite element simulation of the transmittance of the 200 µm long terahertz coplanar waveguide used in the experiment.

The frequency-dependent real and imaginary parts of the impedance of the coplanar waveguide Z_w for the quasi-TEM pulse, calculated using CST studio suite, are shown in Fig. S 12. Within the frequency range (40-500 GHz) relevant for present experiments, both Re[Z] and Im[Z] exhibit a weak frequency dependence. Re[Z] is around 59 Ω and is much larger than Im[Z] (|Im[Z]| < 0.2 Ω). Therefore, in the calculation for the impedance of the K_3C_{60} film, we use a purely real impedance for the waveguide, Z_w=59 Ω.

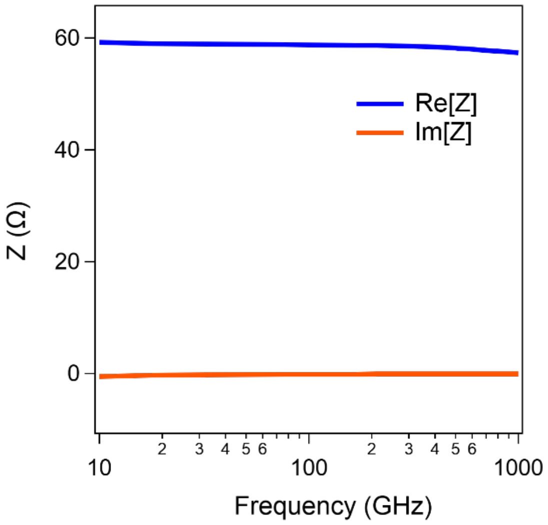

Fig. S 12| Simulated frequency dependence of the real and imaginary parts of the impedance of the terahertz coplanar waveguide used in the experiment.

S3.6: Calibration of peak current value of ultrashort electrical pulse

To calibrate the peak current value of the ultrashort electrical pulse, the net charge of the electrical pulse was collected at one end of the signal line, which was measured using a combination of a transimpedance amplifier and a lock-in amplifier, as shown in Fig. S 13. The peak current value is calculated with the relation:

$$I_{peak} = \frac{1}{2} \cdot \frac{2\sqrt{2}}{1.273} \cdot \frac{V_{Lock-in}}{A_{TIA}} \cdot \frac{1}{f_{rep}} \cdot \frac{1}{\int pulse_{norm}(t)dt} \quad (1)$$

Where I_{peak} is the current peak value, $A_{\text{TIA}}(= 2 \cdot 10^9 \frac{V}{A})$ is the amplification factor of transimpedance amplifier, f_{rep} $(= 50\ kHz)$ is the laser repetition rate, and $\int pulse_{\text{norm}}(t)dt$ ($\sim 1.5 \cdot 10^{-12} s$) is the temporal integral of the normalized electrical pulse. The factor $1/2$ comes from the fact that two equal electrical pulses propagate towards left and right direction. The factor $2\sqrt{2}/1.273$ describes the relation between the net charge amplitude and the root mean square (RMS) amplitude measured at the lock-in amplifier at the chopping frequency. The calculated peak current value of reference pulse (V_1(t)) versus bias voltage is shown in Fig. S 13b. In the main text, the data are plotted versus the peak current value of the transmitted pulse V_2(t), which is $I_{\text{peak}} \cdot \frac{V_{2,\text{peak}}}{V_{1,\text{peak}}}$.

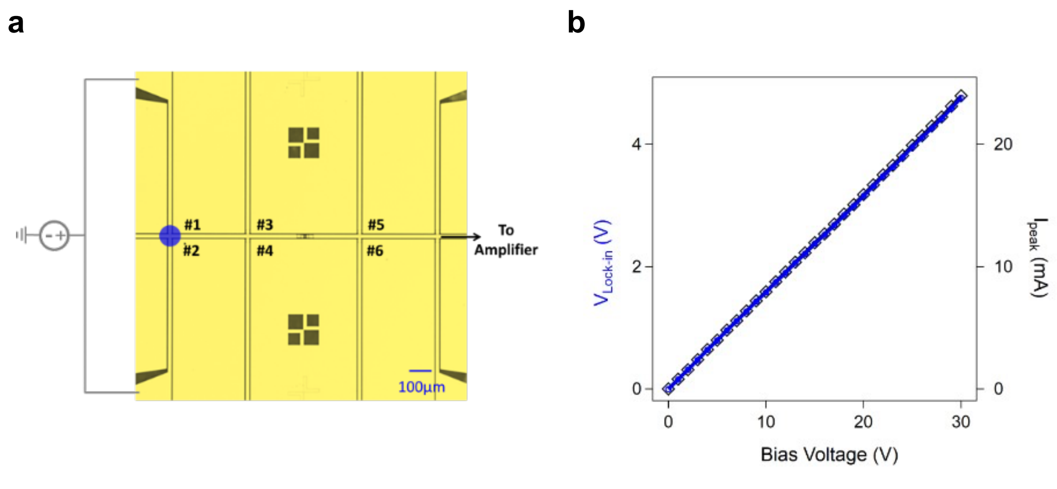

Fig. S 13| Peak current value calibration for the launched electrical pulse. (a) Setup of measurements. (b) The signal from Lock-in amplifier (blue dot) and the calculated peak current (black square) versus bias voltage.

S3.7: Characterization of the K_3C_{60} thin film

The surface morphology of the thin film was checked with atomic force microscopy before doping process. As shown in Fig. S 14, the thin film is polycrystalline with a domain size ~ 100

nm on average. The average domain thickness is estimated to be ~ 20 nm from the surface roughness.

A resistance versus temperature measurement is shown in Fig. S 15, where the resistance increases with increasing temperature, demonstrating the metallic behavior of the K_3C_{60} thin film. This further emphasizes that the photo-induced state with reduced resistance is a non-thermal effect.

To confirm that the thin film was uniformly doped, the superconducting transition (resistance versus temperature) was measured with different bias currents as shown in Fig. S 16. The transition curves become broader with increasing bias current, but do not show a double transition feature, which has been observed as a typical characteristic in inhomogeneous superconductors [4-6]. This is indicative of homogeneous doping in our K_3C_{60} thin film.

To further support the scenario that the K_3C_{60} thin film undergoes a transition from a state that consists of weakly-coupled superconducting grains to a phase-coherent bulk superconductor when cooling through the broad superconducting transition, we plot V versus $(I - I_c)$ with logarithmic scale in Fig. S 17, to get the value of α in the relation $V \propto (I - I_c)^{\alpha}$. The change of α from ~ 1 to 3 when the sample is cooled down from 15 K to 8 K, is consistent with this scenario as shown in previous studies [7, 8]. This two-dimensional crossover behavior ($\alpha = 3$) is attributed to the X-Y network of coupled superconducting domain boundaries [8].

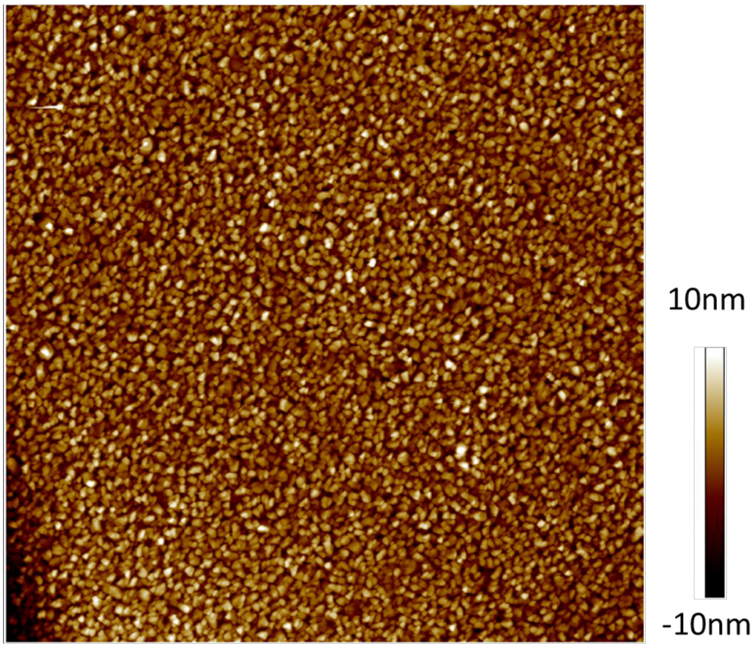

Fig. S 14| A atomic force microscopy image of the K_3C_{60} thin film. The image size is 5x5 µm.

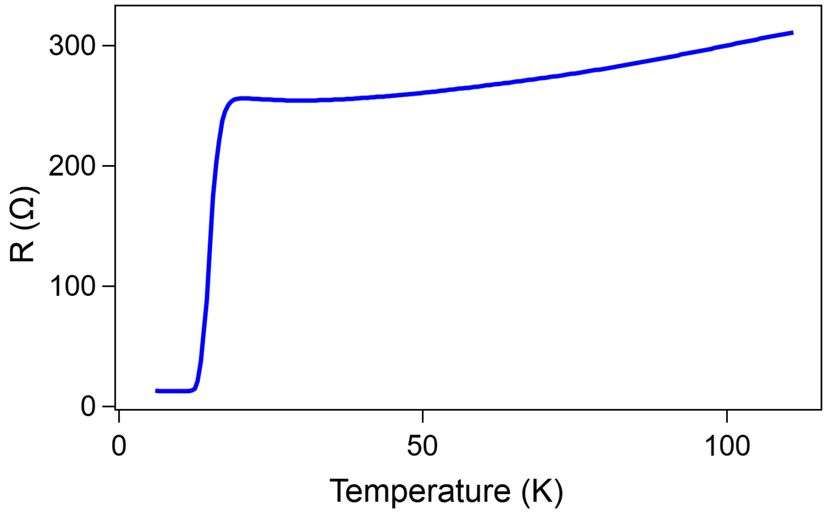

Fig. S 15| Resistance vs temperature measurement, with 1 μA bias current.

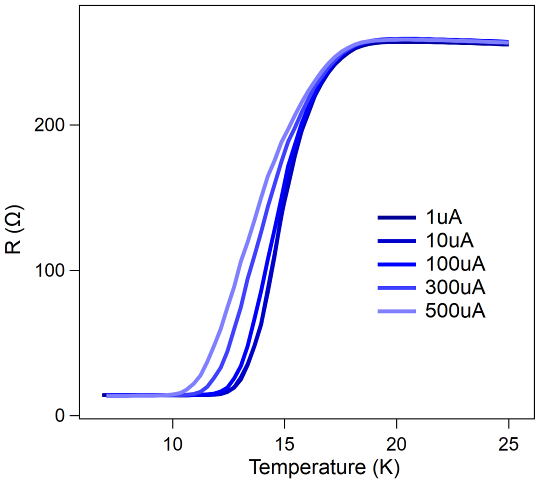

Fig. S 16| Resistance versus temperature measurements near T_c with different bias currents.

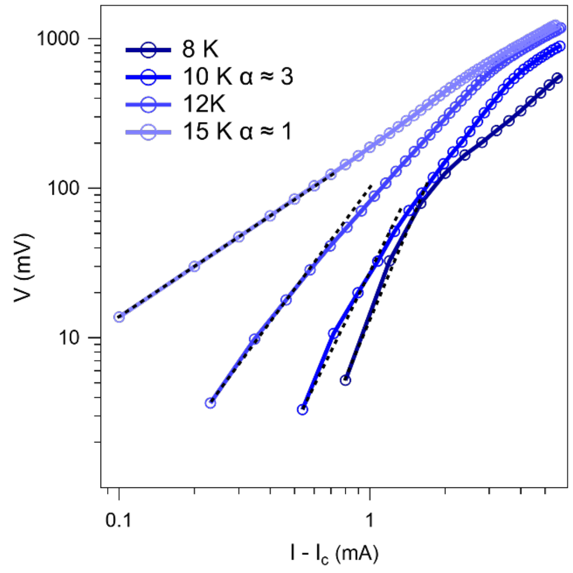

Fig. S 17| Current-voltage characteristics of the K_3C_{60} thin film below T_c.

S4. Data analysis

S4.1: Subtraction of the reflected signal

The raw data of the transmitted pulse $V_2(t)$ at 25 K is shown in Fig. S 18. At ~ 6 ps, there is a second peak in addition to the first peak at ~ 2 ps. While the first peak stems from the transmission of the incoming reference pulse $V_1(t)$, the second peak originates from the double reflection between switches #5,6 and the K_3C_{60} thin film (indicated by the red arrows in Fig. S 18a), because the switches behave like a weak, local disturbance to the propagation of electrical pulse. This assignment is verified by a propagation time analysis as follows. The propagation speed of the electrical pulse in the coplanar waveguide is ~132 μm/ps, as determined by considering $c/\sqrt{\varepsilon_{eff}}$, where c is the speed of light and $\varepsilon_{eff} \approx (\varepsilon_{air} + \varepsilon_{sapphire})/2 = 5.2$ is the effective dielectric constant. The distance between sample and switches #5,6 is 240 μm (see Fig. S18). Given these values, the roundtrip pulse propagation time between the sample and switches #5,6 is ~3.64 ps, consistent with that observed in the experiment (see Fig. S19).

The tail of the transmitted pulse is mixed with the rising edge of the second peak. Without subtraction, as the second peak is from the reflection between the switches and the sample, the transmitted pulse can be expressed as $V_2(\omega) = V_1(\omega) \cdot (\hat{t}_{sample} + \hat{t}_{sample} \cdot \hat{r}_{switch} \cdot \hat{\rho}_{propagation} \cdot (1 - \hat{t}_{sample}))$ and this leads to $\frac{V_2(\omega,T)}{V_2(\omega,20\ K)} = \frac{\hat{t}_{sample}(T)(1+\hat{r}_{switch}\cdot\hat{\rho}_{propagation}\cdot(1-\hat{t}_{sample}(T)))}{\hat{t}_{sample}(20\ K)(1+\hat{r}_{switch}\cdot\hat{\rho}_{propagation}\cdot(1-\hat{t}_{sample}(20\ K)))}$, where $\hat{t}_{sample}, \hat{r}_{switch}, \hat{\rho}_{propagation}$ denote transmission at sample position, reflection at switches #5,6 and propagation along coplanar

waveguide. The calculation of $\hat{t}_{sample}$ is complicated when the exact forms of $\hat{r}_{switch}$ and $\cdot\, \hat{\rho}_{propagation}$ are unknown.

To separate the second peak from the measured data, we fit the rising edge with a Gaussian function (red curve in Fig. S 18b) and subtract it from the raw data. This second peak also affects the measurements at other temperatures. Because the amplitude of this second peak is proportional to both the transmittance and reflectance of the sample, we calculate the rising edge at temperature T using the relation:

$$V_{rise}(t,T) = \frac{V_{2,peak}(T)(1-V_{2,peak}(T))}{V_{2,peak}(25\,K)(1-V_{2,peak}(25\,K))} \cdot V_{rise}(t, 25\,K) \qquad (2)$$

where, $V_{\mathrm{rise}}(t, 25\,K)$ is the fitted rising edge of the second peak at 25 K, and $V_{2,\mathrm{peak}}(T)$ is the peak value of the transmitted pulse $V_2(t)$ (normalized by peak value of $V_1(t)$) at temperature T. There is a temperature dependence associated with the phase shift of the reflected pulse from the sample. To avoid this affecting our analysis, we only subtract the raw data with the rising edge up to a sampling time of 5 ps. The data after subtraction of the rising edge is shown in Fig. S 19. And after subtraction, $\frac{V_2(\omega,T)}{V_2(\omega,20\,K)} = \frac{\hat{t}_{sample}(T)}{\hat{t}_{sample}(20\,K)}$.

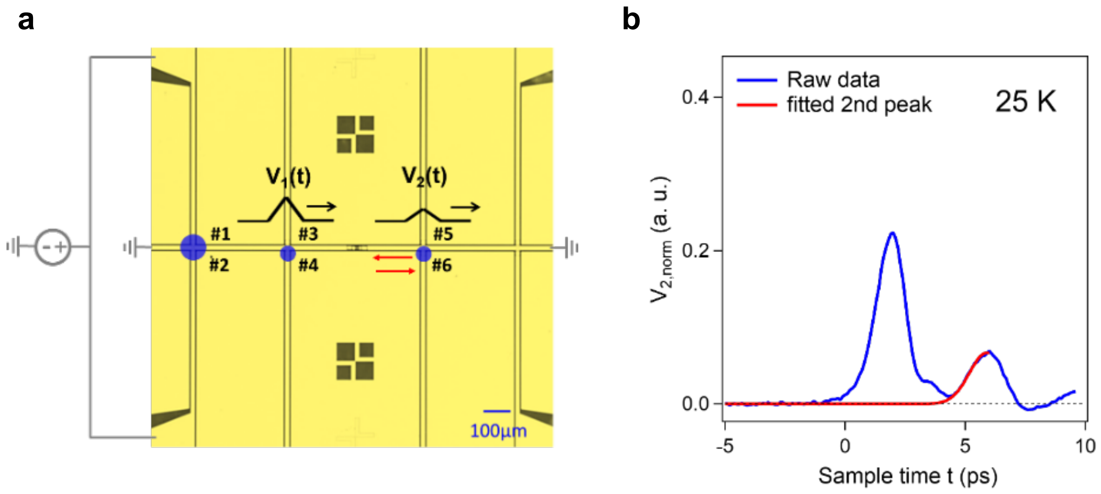

Fig. S 18| Fitting of the second peak, which stems from the reflection between the photo-conductive switches and the sample.

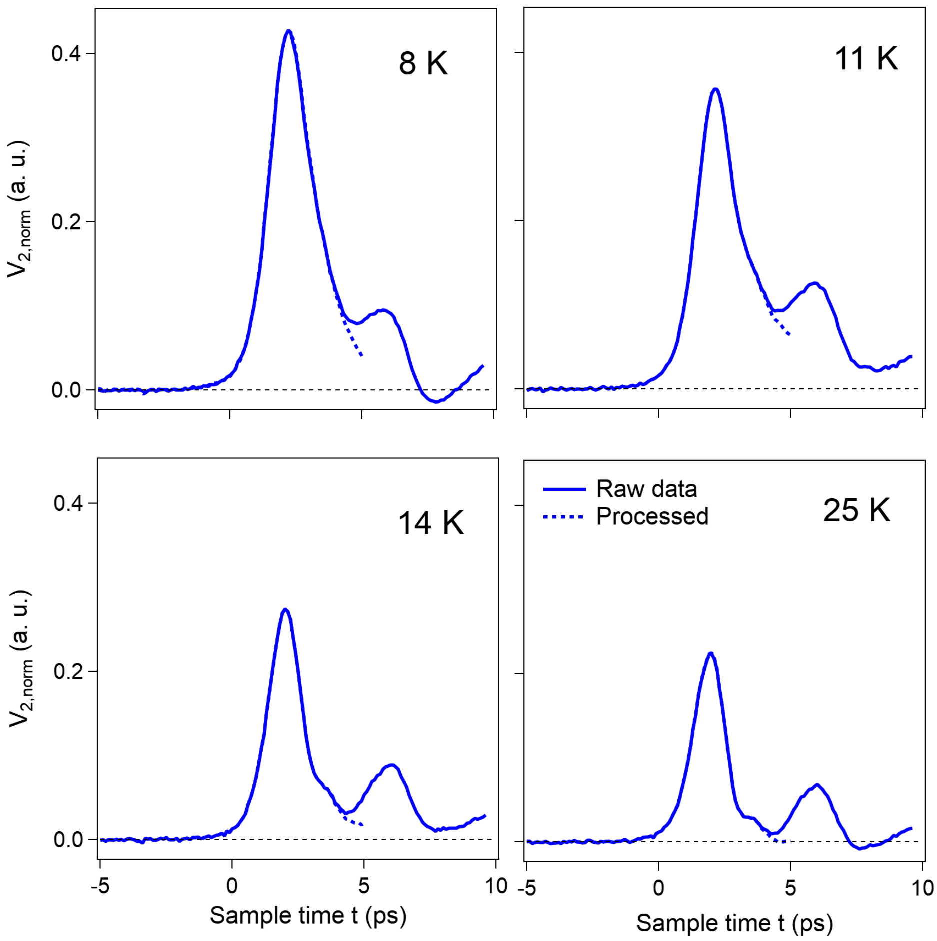

Fig. S 19| Data after subtracting the rising edge of the second peak.

S4.2: Double-exponential fitting of data

For lower temperatures, the data, after subtracting the rising edge of the second peak, terminates abruptly at sampling time 5 ps. In order to extrapolate the pulse, we fit the tail of the data with a double-exponential function. As for the data in which the amplitude drops to zero at 5 ps, the data is extended by setting the value at later sampling times to zero. The quality of the fit and extrapolation of the data with a double-exponential function at lower temperatures is shown in Fig. S 20.

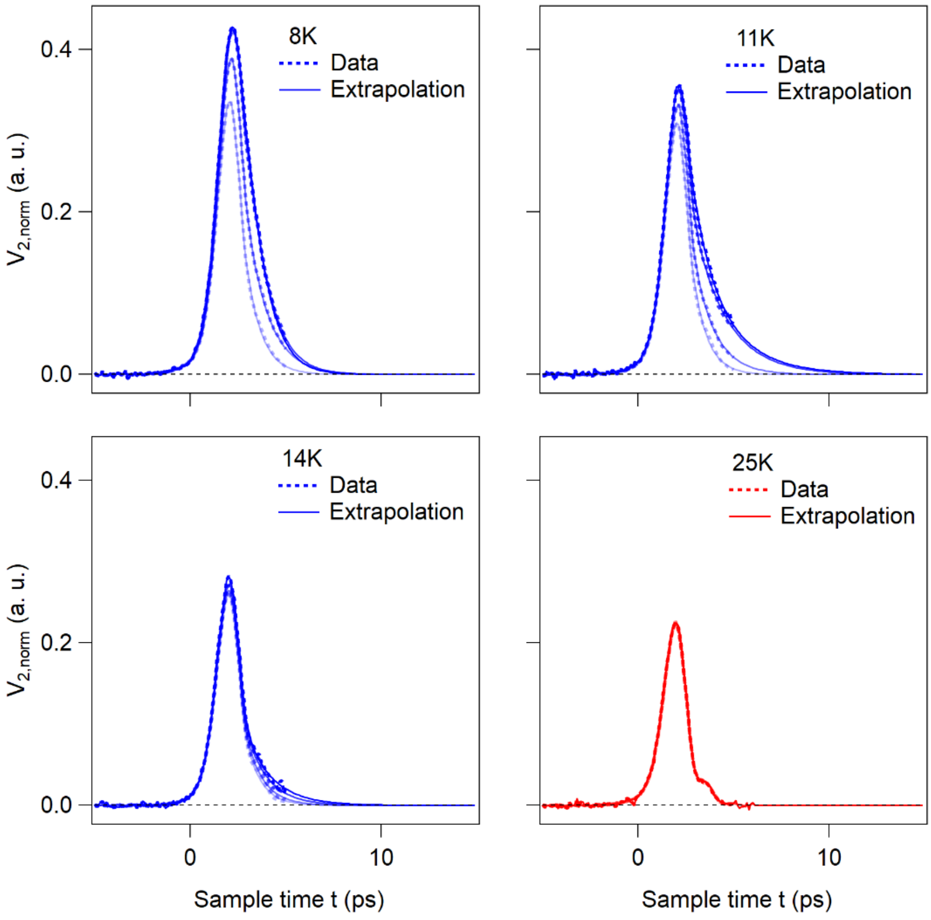

Fig. S 20| Extrapolated data with double-exponential fitting.

The reason why the fit and extrapolation shown in Fig. S 20 works well is as follows. In the electrical network shown below, Z_w denotes the frequency-dependent impedance of the coplanar waveguide and $\hat{Z}$ the impedance of the sample. As shown in Section S3.5, the Z_w can be treated as a pure resistance ~ 59 Ω. The reflectance S_{11} and transmittance S_{21} can be calculated from the relations below:

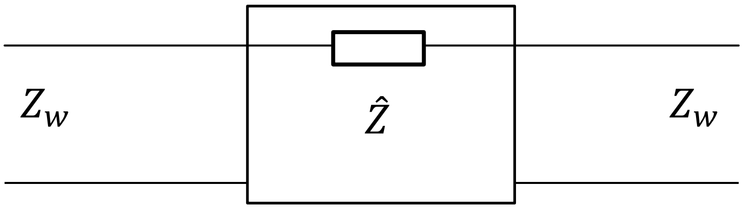

$$S_{11} = \frac{\hat{Z}}{2Z_w + \hat{Z}} \tag{3}$$

$$S_{21} = \frac{2Z_w}{2Z_w + \hat{Z}} \tag{4}$$

When sample is superconducting, we can model the sample with the following two-fluid circuit:

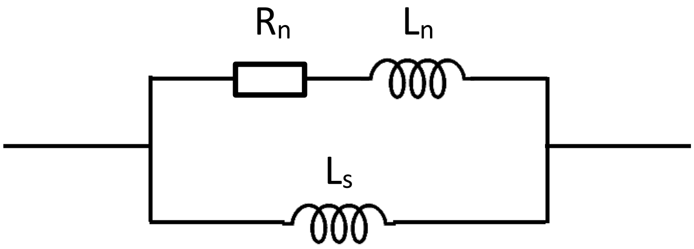

Its effective impedance is (using the Laplace transformation formalism):

$$\begin{aligned}\hat{Z}(s) &= (R_n + sL_n) \parallel (sL_s) \\ &= \frac{(R_n + sL_n) \cdot sL_s}{(R_n + sL_n) + (sL_s)} \\ &= \frac{s \cdot R_n L_s + s^2 \cdot (L_n L_s)}{R_n + s \cdot (L_n + L_s)}\end{aligned} \tag{5}$$

Therefore, the transmittance $S_{21}(s)$ is expressed as:

$$\begin{aligned}S_{21}(s) &= \frac{2Z_w}{2Z_w + \hat{Z}(s)} \\ &= \frac{2Z_w(L_n + L_s) \cdot s + 2Z_w R_n}{(L_n L_s) \cdot s^2 + [2Z_w(L_n + L_s) + R_n L_s] \cdot s + 2Z_w R_n} \\ &= \frac{s + d}{b \cdot s^2 + c \cdot s + d}\end{aligned} \tag{6}$$

Where,

$$b = \frac{L_n L_s}{2Z_w(L_n + L_s)}$$

$$c = 1 + \frac{R_n L_s}{2Z_w(L_n + L_s)}$$

$$d = \frac{R_n}{L_n + L_s}$$

To go back to the time-domain, the inverse-Laplace transformation yields:

$$\mathcal{L}_s^{-1}[S_{21}(s)](t) = a_1 \cdot e^{-t/\tau_1} + a_2 \cdot e^{-t/\tau_2} \tag{7}$$

Where,

$$1/\tau_1 = (\frac{c}{2b} + \frac{\sqrt{c^2 - 4bd}}{2b})$$

$$1/\tau_2 = (\frac{c}{2b} - \frac{\sqrt{c^2 - 4bd}}{2b})$$

$$a_1 = \frac{1}{\sqrt{c^2 - 4bd}}(\frac{\sqrt{c^2 - 4bd} + c}{2b} - 1)$$

$$a_2 = \frac{1}{\sqrt{c^2 - 4bd}}(\frac{\sqrt{c^2 - 4bd} - c}{2b} + 1)$$

The parameter values in the above equations are given by the relations: $R_n = \frac{R_{n0}}{n_n(T)}$, $L_n = \frac{L_{n0}}{n_n(T)}$, and $L_s = \frac{L_{s0}}{n_s(T)}$, where the Cooper pair density ratio $n_s(T) = 1 - (\frac{T}{T_c})^4$, the normal carrier density ratio $n_n(T) = 1 - n_s(T)$, the resistance of normal state $R_{n0} = 250\ \Omega$ from experimental measurement, and the kinetic inductance of the normal state $L_{n0} = \tau \cdot R_{n0} = 55\ pH$ (τ from Ref. [9]). $L_{s0} = L_{n0} = 55\ pH$ is the kinetic inductance of the superconducting state at 0 K, assuming that the effective mass of a Cooper pair is double the mass of a normal carrier.

The physical meaning of these two timescales $\tau_1$and $\tau_2$in the two exponentials are associated with the decay times of the two inductive components L_n and L_s, which originate from two types of carriers (normal carriers and Cooper pairs) as shown in the circuit model above. Because the lifetime of the photo-induced effect (see Fig. S24) is much longer than our sampling time range ~20 ps, we assume the fractions of normal carriers and Cooper pairs in the photo-induced state stay constant during the measurements. We consider two extreme cases below where there are either only normal carriers or only Cooper pairs, to clarify this assignment.

In the first case when the superconducting fraction is zero ($n_s/n_{tot} = 0$), L_s is infinite, as $L_s \propto 1/n_s$. Applying the equations above, we get $\tau_1 = L_n/(2Z_w + R_n)$ and $\tau_2 = L_s/(2Z_w + R_n) = \infty$, which are the decay times for the two resistor-inductor (RL) circuits. In the second case when the superconducting fraction is maximum ($n_s/n_{tot} = 1$), L_n is infinite. With the same formula, we obtain $\tau_1 = L_n/(2Z_w + R_n) = \infty$ and $\tau_2 = L_s/(2Z_w)$, which are again the corresponding decay times for the RL circuits. When there are both normal carriers and Cooper pairs, the analytical formula shown above would be more complicated, because there is also current flow between these two inductances in parallel, but in general the above explanation gives the physical meanings of these two decay timescales. At 8 K, from the calculation, we get $\tau_1 = 0.14\,ps$ and $\tau_2 = 0.52\,ps$, which are consistent with the fitting parameter values used for the data measured at 8 K.

S5. More data

S5.1: Measurements with a nanosecond pulse generator

To bridge the gap between the nonlinear features of superconducting state observed with DC and picosecond current, we applied nanosecond voltage pulses to the K_3C_{60} thin film from outside the chip, and monitored how the resistance of K_3C_{60} changed at 8 K, with increasing the pulse duration and amplitude. The electronic setup is shown in Fig. S 21a, in which a pre-resistor (100 Ω) was placed before the sample in order to monitor the current flowing through the sample during the measurements. The voltages across the pre-resistor plus sample and across only the sample were monitored and recorded using an oscilloscope. Fig. S 21b and c show the voltage time-traces across the pre-resistor plus sample (red) and across only the sample (blue), for different durations of the applied voltage pulse. With a small voltage pulse bias (0.5 V), oscillations from the sample's inductive response are observed at early times and then the voltage becomes almost zero with a small contribution from the contact resistance. With a larger voltage pulse bias (6 V), the voltage across the sample starts to increase at early times and reaches equilibrium at later times in the 1 µs case. In the 150 ns case, the voltage pulse bias is gone before the voltage across the sample can stabilize.

The data points in the final 50 ns of the voltage pulse were used to calculate the bias current of the sample and subsequently the resistance. The resistance versus bias current curves measured at 8 K for different voltage pulse durations are summarized in Fig. S 22. As shown, for all pulse durations, the resistance starts to increase when the current increases above the same critical value. The change in resistance becomes steeper with longer pulse duration, which is consistent with what has been observed for DC and picosecond transport measurements.

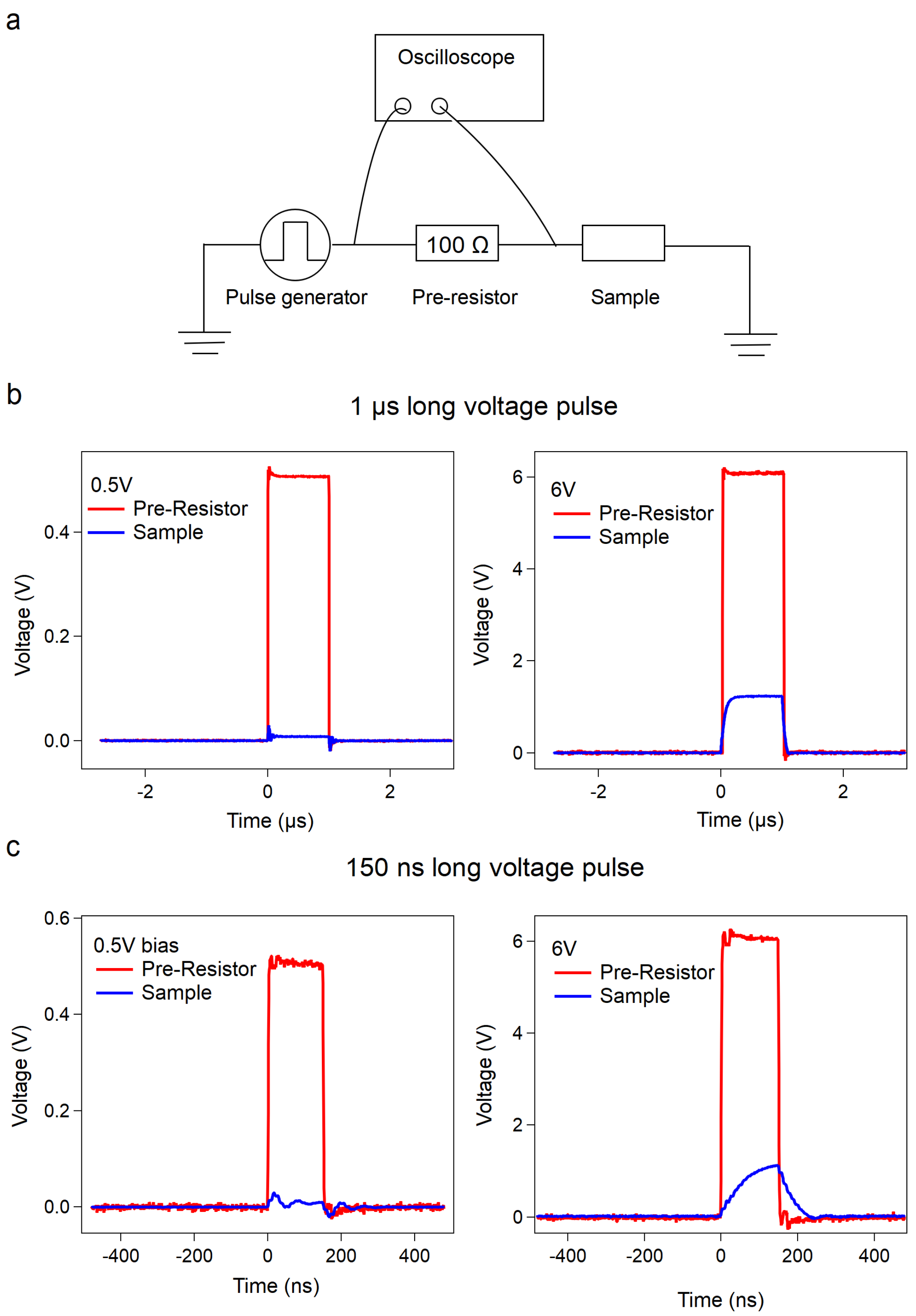

Fig. S 21| Time-trace of the voltage across the pre-resistor + sample (red) and across only the sample (blue) when the sample was biased using an external pulse generator. (a) Schematics of the measurement. The temperature of sample is kept at 8 K. (b) Two time-traces of the voltage potential for a 1 μs long voltage pulse with 0.5 V and 6V amplitude. (c) Same as (b) but for a 150 ns long voltage pulse.

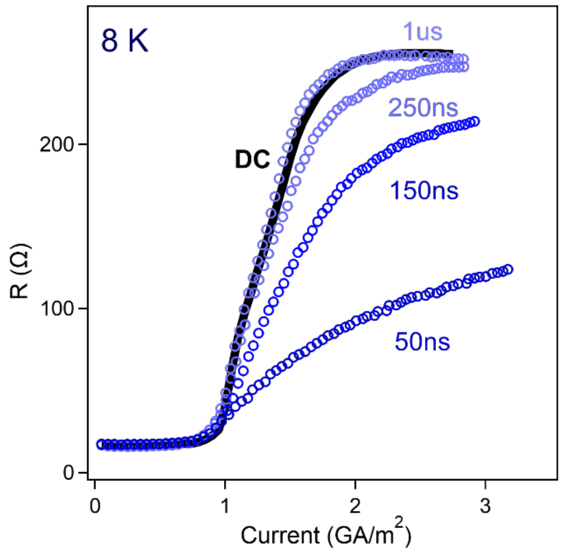

Fig. S 22| Resistance versus bias current for different voltage pulse durations. The temperature of sample is kept at 8 K.

S5.2: Ultrafast transport measurements

S5.2.1: Photo-induced state below and above T_c

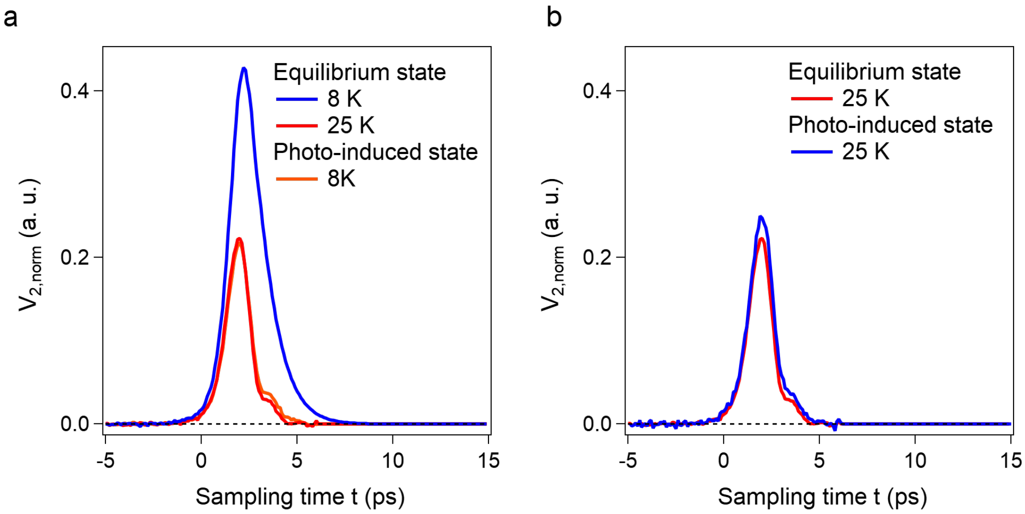

Fig. S 23| Response of the photo-induced state at 8 K and 25 K. The fluence of the mid-IR pulses is 4mJ/cm² for both cases.

Below T_c, the superconductivity is destroyed upon mid-IR excitation, as the photon energy is larger than the double of superconducting gap size. Although it is not yet fully understood why the effect of mid-IR excitation is different below and above T_c, the difference in properties of the initial states is a likely candidate.

The response of the photo-induced state at 8 K is shown in Fig. S 23a, which is similar to the response of the equilibrium state at 25 K. This can be understood, as the mid-IR excitation breaks the Cooper pairs into normal carriers. The response of the photo-induced state at 25 K is also shown (Fig. S 23b). Because of the formation of granular superconductivity compared with the bulk superconductivity at equilibrium 8 K, the transmittance is not as high as the equilibrium 8 K.

We also studied the lifetime of the photo-induced state at 8 K and 25 K. As shown in Fig. S 24, the lifetime of photo-induced state at 8 K is much longer than 2 ns, which is related with the dissipation of the energy injected by the mid-IR pulses into the K_3C_{60} thin film. On the other hand, the lifetime of the photo-induced state at 25 K is around 2 ns. This also makes the extraction of impedance of the photo-induced state at 50 GHz possible.

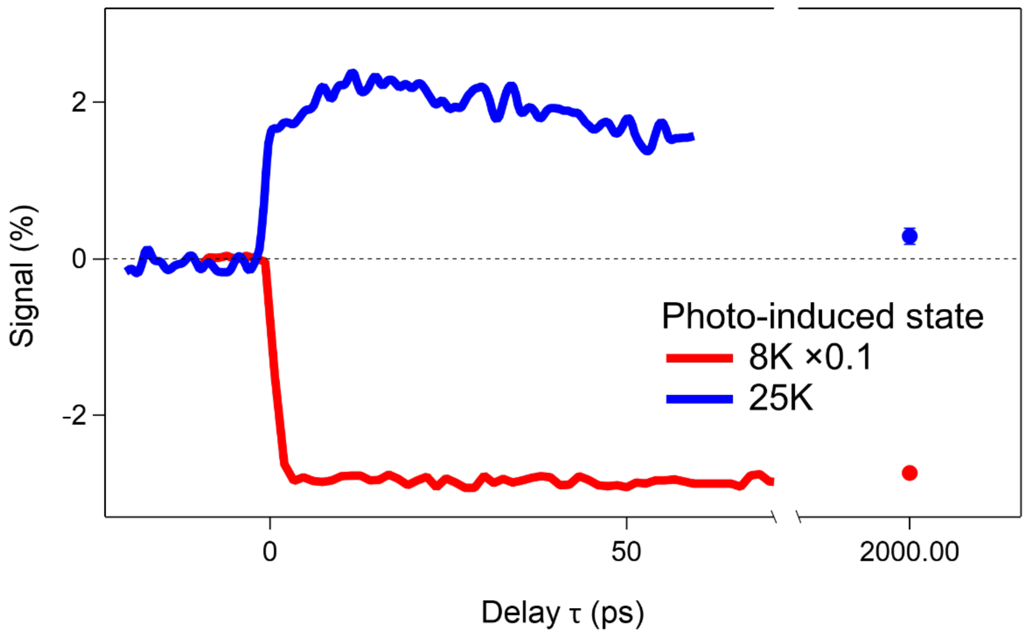

Fig. S 24| Lifetime of the photo-induced state at 8 K and 25 K.

S5.2.2: Fluence dependence

More fluence dependent data is shown in Fig. S 25. The transmittance at low frequency increases with stronger mid-IR pump and saturates at around 4 mJ/cm^2 (Fig. S 25a) where the drop of Re[Z] also saturates (Fig. S 25b). The nonlinear behavior also gradually develops with increasing mid-IR pump fluence (Fig. S 26).

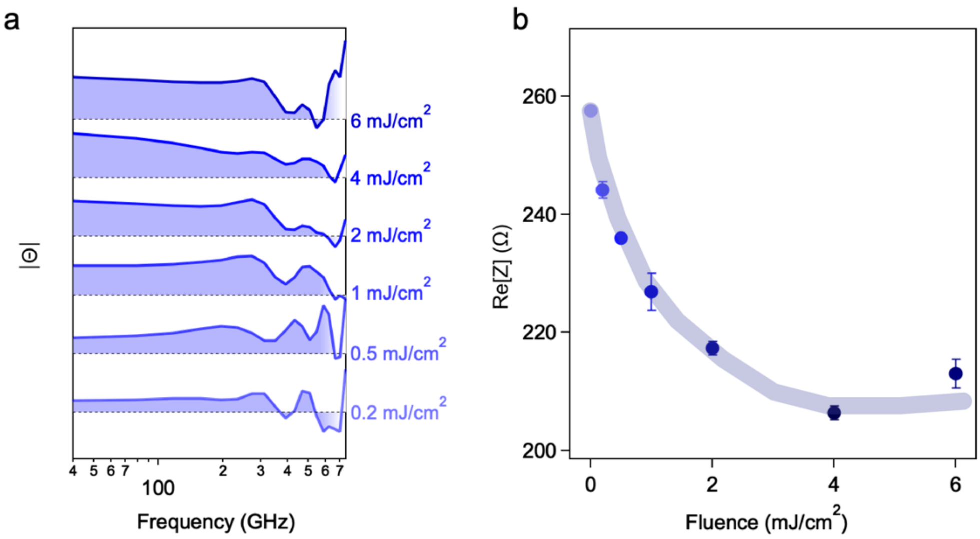

Fig. S 25| Fluence dependent measurements of the photo-induced state at 25 K. (a) Normalized transmittance $|\Theta|$ for different mid-IR fluences. The peak current density of the picosecond electrical pulse is 0.1 GA/m^2. The curves are shifted vertically for clarity and the dashed line indicates unity. (b) Calculated $Re[Z]$ at 50 GHz versus fluence. The error bars shown are defined as standard errors.

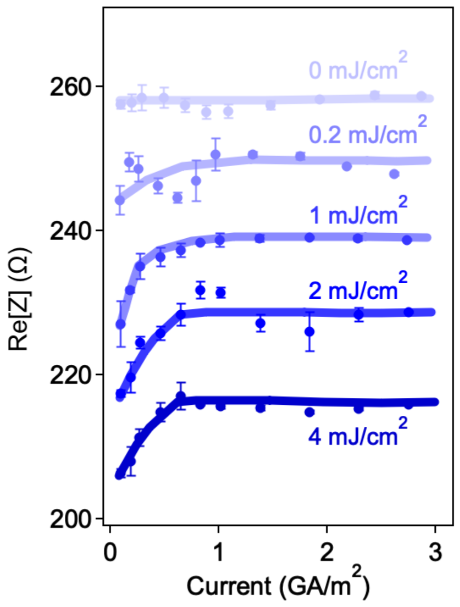

Fig. S 26/ Fluence dependence of nonlinear transport behavior in photo-induced state. The peak current density of the picosecond electrical pulse is 0.1 GA/m^2. The error bars shown are defined as standard errors.

S6. Simulation

S6.1: Time-domain simulation

The time-trace of the transmitted electrical pulse $V_2(t)$ was simulated with finite-element time-domain method using CST studio suite software, with the measured $V_1(t)$ and the optical conductivity of K_3C_{60} extrapolated from data of Ref. [9] as input parameters. As shown in Fig. S 27, the data and simulation are alike.

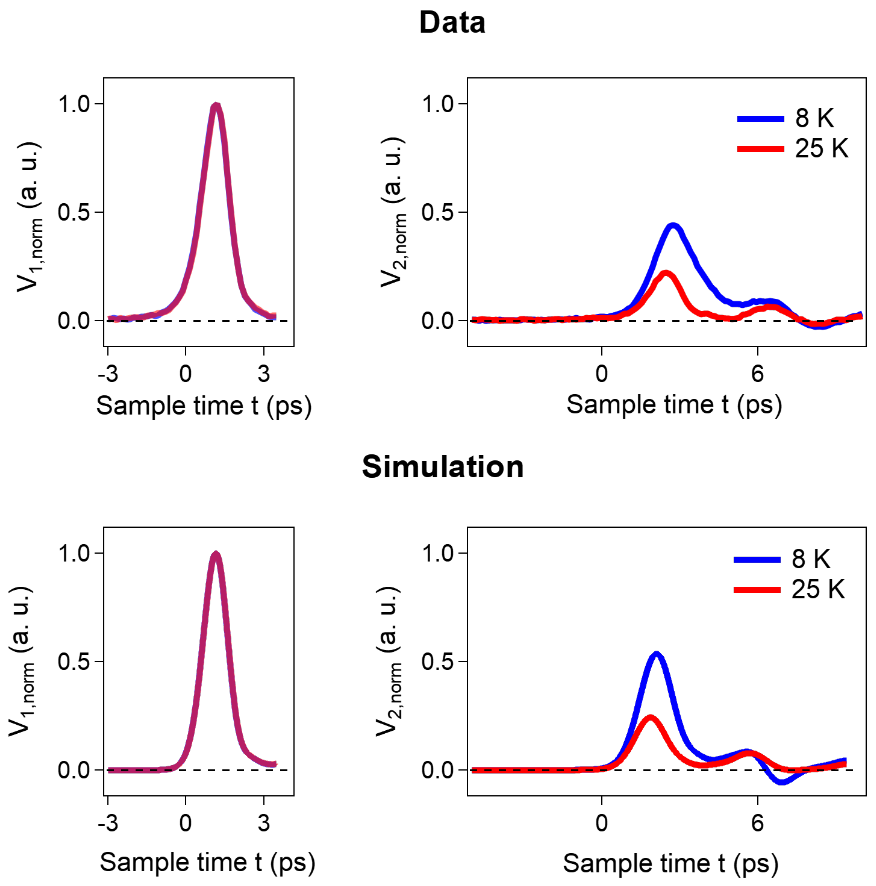

Fig. S 27| Comparison of measured data and finite-element time-domain simulation of the transmitted electrical pulse.

S6.2: Frequency-domain calculation

To explain the dip at frequency ~ 700 GHz observed in Fig. 2b, we calculated the normalized transmittance at 8 K in the frequency domain with Qucs Studio software by treating sample with two fluid circuit model used in Section S4.2. As shown in Fig. S 28, without capacitive coupling, the normalized transmittance of the superconducting state at 8 K is always larger than 1. This is contrasted with the case with a small capacitance contribution in parallel with the sample, in which the response could well reproduce the dip observed in the experiment. The value of the capacitance being used in the calculation is ~ 0.7 fF, which is congruous with the capacitance value between the two contacts of K_3C_{60} film calculated with the method from Ref. [10].

To simulate the temperature dependence of |Θ|, we need to consider the resistive response of the weak links between superconducting grains at intermediate temperatures. The detailed physical origin is discussed in Section S6.3. Here the measured resistance values of the sample (shown in Fig. 2 and Fig. S 16) are used as the values of the effective resistance R_{WL}, contributed by the weak links. The rest superconducting grains are treated with two-fluid circuit model. The calculated normalized transmittances at different temperatures are shown in Fig. S 29b, which could well reproduce the data shown in Fig. S 29c.

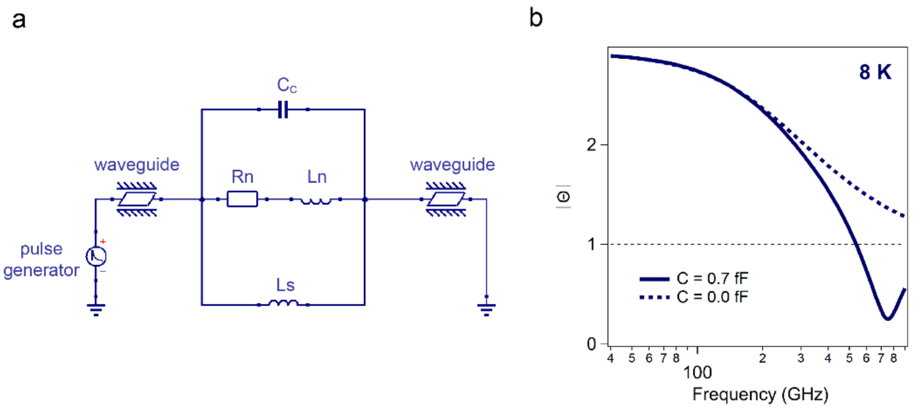

Fig. S 28| Transmittance calculation with Qucs Studio software.

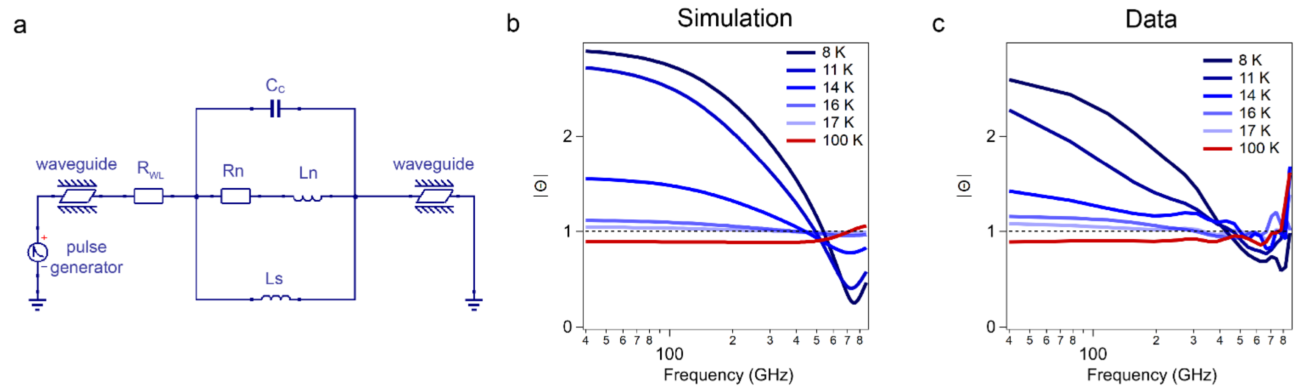

Fig. S 29| Transmittance calculation with Qucs Studio software, considering the resistive response of the weak links between the superconducting grains.

S6.3: Critical current simulation

It has been known that the polycrystalline sample has a broader superconducting transition compared with a single-crystal sample, for the same superconducting material [8]. This has been attributed to the weak coupling between superconducting grains at intermediate temperatures [7, 8]. When the coupling energy between the superconducting grains ($\hbar I_c(T)/2e$, i.e., the energy cost for a phase slip) is comparable with the thermal fluctuation

energy ($k_B T$), the phase slip can be thermally activated at weak links and the polycrystalline sample shows resistive response, despite the superconducting grains. Calculations can be done to check whether the K_3C_{60} film falls into this range. From the AFM image shown in Fig. S 14, we estimate the average K_3C_{60} grain size is 100 nm(W) x 100 nm(L) x 20 nm(T). We can therefore consider the 20 um(W) x 20 um(L) x 100 nm (T) sample to be a 200(W) x 200(L) x 5(T) mesh of grains, which are connected by weak links. A cross-section of this mesh contains 1000 weak links along the current direction. From the DC transport measurement shown in Fig. S 30a, we can make a first estimation of the low-temperature critical current $I_c(0\ \mathrm{K})$ of a single weak link of $3\ \mathrm{mA}\ /\ 1000\ =\ 3\ \mu\mathrm{A}$. Based on this, the coupling energy of a single weak link is ~ 4 meV at 10 K (given $I_c(T) = I_c(0\ \mathrm{K}) * (1 - (T/T_c)^{1.5})$, which is comparable with the corresponding thermal fluctuation energy ~ 1 meV. This estimation shows that the competition between the coupling of the superconducting grains and the thermal fluctuation can affect the transport properties of the K_3C_{60} thin film.

To simulate the effect of thermally activated phase slip on sample's transport properties, we describe the $V - I$ relation of a single weak link with the equations of motion for Josephson junction [11-13], which are shown below:

$$d\theta/dt = 2eV/\hbar \tag{8}$$

$$C\ dV/dt = I - I_c(T)sin\theta - \frac{V}{R} + \tilde{L}(t) \tag{9}$$

Equation (1) is the Josephson condition relating θ, the phase difference of the order parameter of two adjacent superconducting grains and V, the potential difference. Equation (2) describes the condition of conservation of charge. C is the capacitance of the junction; $I_c(T)$ is the maximum Josephson current at temperature T in the absence of noise; R is the resistance of the junction which is approximately the resistance in the normal state; $\tilde{L}(t)$ is the fluctuating noise current, which is thermal and given by $\langle \tilde{L}(t+\tau)\tilde{L}(t) \rangle = 2R^{-1}k_B T$. To get the $V - I$ relation of the sample, we consider that the sample has a 200(W) x 200(L) x 5(T) mesh of weak links as described before.

In the calculation, we estimate the value of C ~ $10^{-17}F$ from $\varepsilon_0 \cdot S/d$, where ε_0 is the vacuum electrical permittivity; S is the cross-section area of the weak link (100 x 20 nm^2); d is the average distance between two adjacent superconducting grains, where we use 1 nm as a lower limit, because the domains are actually loosely touched as shown in the AFM image (Fig. S 14). Using smaller values of C does not change the calculation results, as the equations of motion already fall into the overdamped region as discussed in Refs. [11-13], which is also confirmed by our calculation using a smaller capacitance value. Other than the first estimation ~ 3 μA for $I_c(0\ K)$ of a single weak link, we need to use 6 μA to reproduce the measurements. This is because the phase-dependent supercurrent has been partially washed out by the thermal fluctuations [14]. The comparison between simulations and measurements for DC transport is shown in Fig. S 30. The experimentally observed features, including the finite resistance at small bias current and the vanishing critical current at intermediate temperatures, could be well reproduced. The steeper increase of the

resistance in the measurements is presumably due to the heating effect, which becomes stronger when the sample gets resistive and the sample transits faster into the normal state, which is not included in the equations of motion for a weak link introduced above.

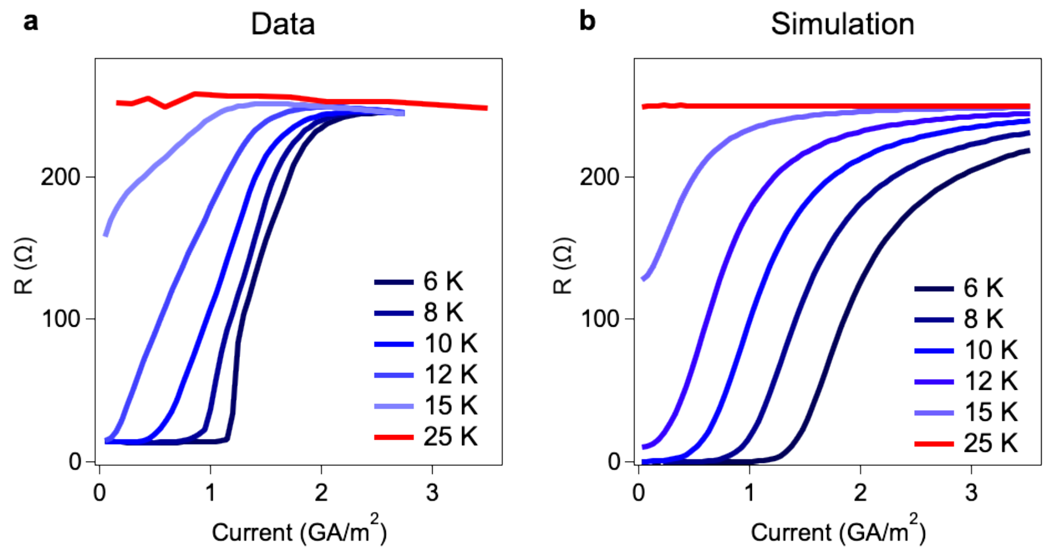

Fig. S 30| Comparison between the data and calculations using equations of motion for Josephson junction.

References:

1. Kozich, V., A. Moguilevski, and K. Heyne, *High energy femtosecond OPA pumped by 1030nm Yb:KGW laser.* Optics Communications, 2012. **285**(21): p. 4515-4518.
2. Haddon, R.C., et al., *Electrical resistivity and stoichiometry of K_xC_{60}, Rb_xC_{60}, and Cs_xC_{60} films.* Chemical Physics Letters, 1994. **218**(1): p. 100-106.
3. Hesper, R., et al., *Photoemission evidence of electronic stabilization of polar surfaces in K_3C_{60}.* Physical Review B, 2000. **62**(23): p. 16046-16055.
4. Gerber, A., et al., *Double-peak superconducting transition in granular L-M-Cu-O(L=Pr,Nd,Sm,Eu,D; M=Ce,Th) superconductors.* Physical Review Letters, 1990. **65**(25): p. 3201-3204.
5. Early, E.A., et al., *Double resistive superconducting transition in $Sm_{2-x}Ce_xCuO_{4-y}$.* Physical Review B, 1993. **47**(1): p. 433-441.
6. Jardim, R.F., et al., *Granular behavior in polycrystalline $Sm_{2-x}Ce_xCuO_{4-y}$ compounds.* Physical Review B, 1994. **50**(14): p. 10080-10087.
7. Abraham, D.W., et al., *Resistive transition in two-dimensional arrays of superconducting weak links.* Physical Review B, 1982. **26**(9): p. 5268-5271.
8. England, P., et al., *Granular superconductivity in $R_1Ba_2Cu_3O_{7-\delta}$ thin films.* Physical Review B, 1988. **38**(10): p. 7125-7128.

9. Degiorgi, L., et al., *Optical properties of the alkali-metal-doped superconducting fullerenes: K_3C_{60} and Rb_3C_{60}.* Physical Review B, 1994. **49**(10): p. 7012-7025.
10. Naghed, M. and I. Wolff, *Equivalent capacitances of coplanar waveguide discontinuities and interdigitated capacitors using a three-dimensional finite difference method.* IEEE Transactions on Microwave Theory and Techniques, 1990. **38**(12): p. 1808-1815.
11. Ambegaokar, V. and B.I. Halperin, *Voltage Due to Thermal Noise in the dc Josephson Effect.* Physical Review Letters, 1969. **22**(25): p. 1364-1366.
12. Falco, C.M., W.H. Parker, and S.E. Trullinger, *Observation of a Phase-Modulated Quasiparticle Current in Superconducting Weak Links.* Physical Review Letters, 1973. **31**(15): p. 933-936.
13. Falco, C.M., et al., *Effect of thermal noise on current-voltage characteristics of Josephson junctions.* Physical Review B, 1974. **10**(5): p. 1865-1873.
14. Tinkham, M., *Introduction to Superconductivity*. 2004: Dover Publications.
